# Is local trait variation related to total range size of tropical trees?

**DOI:** 10.1371/journal.pone.0193268

**Published:** 2018-03-07

**Authors:** Eduardo Chacón-Madrigal, Wolfgang Wanek, Peter Hietz, Stefan Dullinger

**Affiliations:** 1 Department of Botany and Biodiversity Research, University of Vienna, Vienna, Austria; 2 Escuela de Biología, Universidad de Costa Rica, San José, Costa Rica; 3 Department of Microbiology and Ecosystem Science, University of Vienna, Vienna, Austria; 4 Institute of Botany, University of Natural Resources and Life Sciences, Vienna, Austria; INRA—University of Bordeaux, FRANCE

## Abstract

The reasons why the range size of closely related species often varies significantly have intrigued scientists for many years. Among other hypotheses, species with high trait variation were suggested to occupy more diverse environments, have more continuity in their distributions, and consequently have larger range sizes. Here, using 34 tree species of lowlands tropical rainforest in southern Costa Rica, we explored whether inherent trait variability expressed at the local scale in functional traits is related to the species’ total geographical range size. We formed 17 congeneric pairs of one narrow endemic and one widespread species, sampled 335 individuals and measured eight functional traits: leaf area, leaf thickness, leaf dry matter content, specific leaf area, leaf nitrogen content, leaf phosphorus content, leaf nitrogen to phosphorus ratio, and wood specific gravity. We tested whether there are significant differences in the locally expressed variation of individual traits or in multidimensional trait variance between the species in congeneric pairs and whether species’ range size could hence be predicted from local trait variability. However, we could not find such differences between widely distributed and narrow range species. We discuss the possible reasons for these findings including the fact that higher trait variability of widespread species may result from successive local adaptations during range expansion and may hence often be an effect rather than the cause of larger ranges.

## Introduction

Even closely related species often vary in range size by orders of magnitude [1]. The reasons of this variation have intrigued scientists for many years [1,2]. Nevertheless, why some species are narrowly distributed endemics, while other related species have spread widely is still a puzzling question with probably complex causation [3–6]. Species can, for example, occupy a narrow range because they have not had the chance to disperse after a range collapse following e.g. climatic changes [7], are evolutionary young [1], limited to isolated places like oceanic islands or mountain peaks [8], or adapted to rare habitat types [9]. However, many species are range-restricted even without (evident) geographic barriers limiting their distributions [10–12], suggesting that factors other than dispersion are involved in shaping their ranges.

Biological traits determine the ecological niches of species and hence control the composition of ecological communities along environmental gradients [13–15]. Indeed, trait variation across geographical ranges of a species often co-varies with components of the physical environment such as soil and climate, likely reflecting environmental filtering of particular trait values [16,17]. As a corollary, high intra-specific variation in relevant functional traits, whether due to genetic diversity or phenotypic plasticity, should be associated with broad environmental tolerance and / or the ability to exploit a greater variety of resources. These attributes should, in turn, allow species to occupy more diverse environments and thus, eventually, larger ranges [4,6,18,19]. However, intra-specific trait variation is often geographically structured [20–22], among other things as a result of the adaptation of individual populations to the new environments they face with range expansion [23–25]. It is thus unclear whether trait variability is actually a determinant or merely a consequence of range size [6]. In other words, endemic species may be endemic because they have lower trait variability than their widespread relatives, or they may have lower trait variability because they are endemic. This question cannot be resolved by comparing trait variation of narrow and wide range species across their entire respective ranges. However, if geographical area is fixed, the range of environmental variation is similar between the species to be compared. In addition, if we focus on regional population only in such a comparison, continued gene flow is more likely to restrict the effects of local adaptation on intra-specific trait variability for the species to be compared [26]. In such a case, higher trait variability in widespread species would hence actually indicate that this variability is a driver rather than a consequence of range size differences.

Here, we compare intra-population variability in functional traits among congeneric pairs of endemic and widespread tree species that co-occur in a restricted region of southern Costa Rica. Tropical tree species offer an appropriate system for studying these questions because they show significant variation in range sizes [27] as well as pronounced inter- and intraspecific variation in functional traits [14]. Nevertheless, the relationship between range size and trait variation has rarely been considered in this group of species [28]. We explore the variability in leaf area, leaf thickness, leaf dry matter content, specific leaf area, leaf nitrogen content, leaf phosphorus content, leaf nitrogen-to-phosphorus ratio and wood specific gravity. These traits have been shown to be strongly related to the economic spectrum of plants [29,30], some of them have also been used as predictors of tolerance to abiotic and biotic stressors such as drought, nutrient-poor soils, shade, fire and competition, and vary across environmental gradients [31–38]. Following the rationale outlined above, we expect that widespread species will be more variable in individual functional traits and in multivariable trait space than narrow range species. We use congeneric pairs of wide- and narrow-range species in our comparison to exclude confounding phylogenetic constraints on functional trait variability [39].

## Materials and methods

### Study site

**Fig 1:**
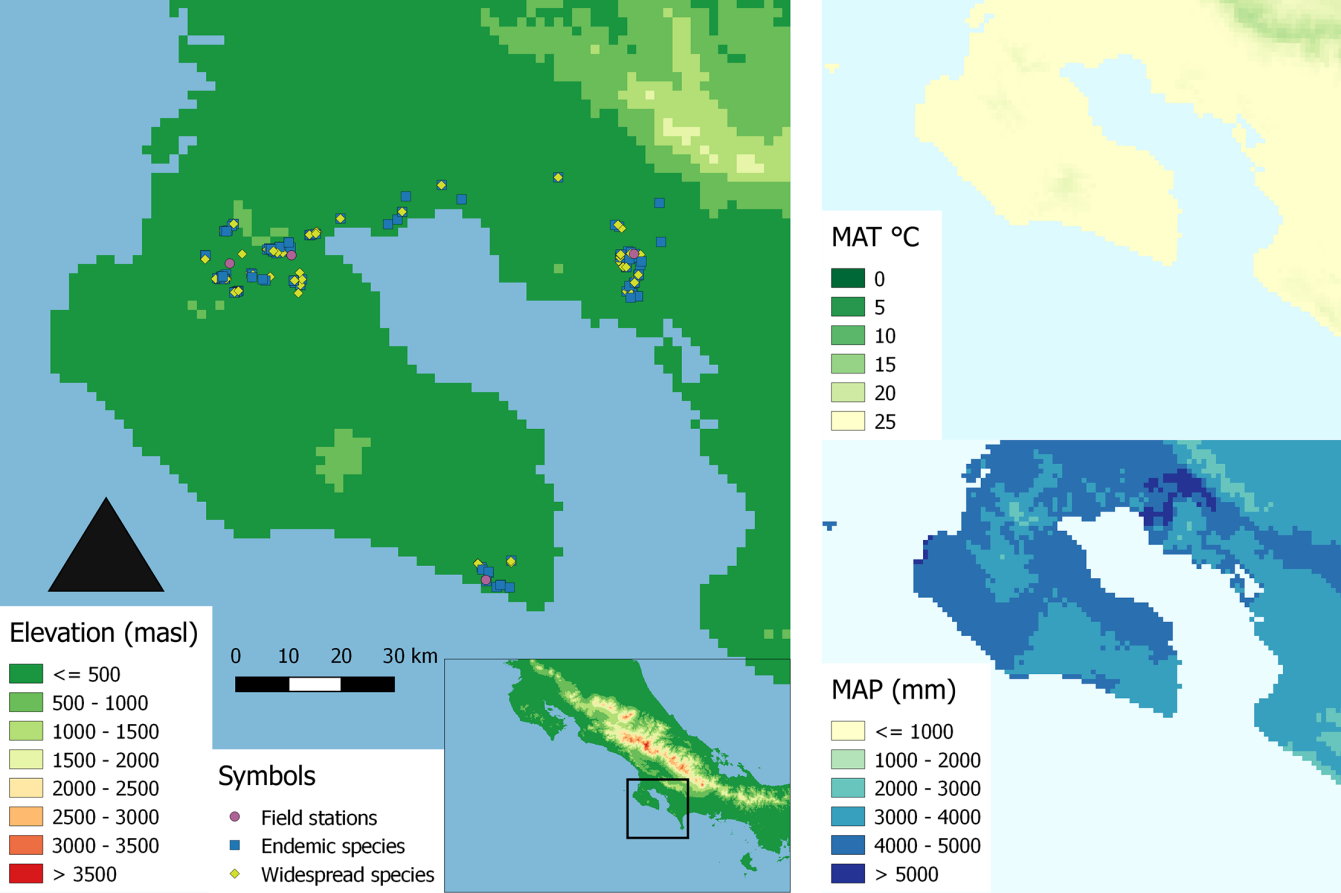
Study area and sampling sites of endemic and widespread species in southeastern Costa Rica (Peninsula de Osa and Golfo Dulce). MAT: Mean annual temperature, MAP: Annual precipitation sum, both according to Hijmans et al. (2005) [40].

Sample collection was allowed under INV-ACOSA-018-14 permission granted by SINAC (Sistema Nacional de Áreas de Conservación, Costa Rica).We worked on the Peninsula de Osa and Golfo Dulce area of southern Costa Rica, in the surroundings of four field stations (8°16'-8°55' N, 83° 4'-83°47 W, Fig 1). Rainfall in the region is between 2800 and 5400 mm/year [40] and mean annual temperature is c. 27°C in the lowlands. There is a short dry season between January and March with occasional rains. On average, 90% of the rain falls between April and December (Fig 1).

High diversity and high levels of endemism characterise the region where more than 2700 vascular plant species have been recorded [41] of which approx. 150 are endemics [42]. The area is particularly recognised for its high richness of trees and palms, with ca. 750 species of trees and 47 species of palms [43]. Floristic affinities are strongest with South American lowland rainforests, especially the northwest of South America [44].

A complex geological history has formed the region since the Late Cretaceous resulting in a landscape with mountains deeply incised by river valleys, hills, terraces, plains and swamps [45]. The causes of speciation and endemism in the region are difficult to disentangle [45]. Compared with the surroundings, the region has a distinct climate because the Talamanca Cordillera towards the North, with mountains as high as 3820 m, creates a vortex effect that increases precipitation and decreases rainfall seasonality [46]. The wetter conditions may have attenuated the climatic fluctuations of the Late Pleistocene [47] and thus probably enhanced chances of in-situ survival for species of the regional flora [9]. Among soils, Ultisols highly weathered and poor in phosphorus are predominant. Alluvial deposits from the Quaternary created the plains and valleys which are dominated by Inceptisols richer in phosphorus [48].

### Species studied

We selected 34 tree species from 14 genera (three genera with each two endemics and two widespread species, and 11 genera with each one endemic and one widespread species) and grouped them into 17 pairs of congeneric species, randomly selecting the pairs in the three genera with four species (Table 1). We note, however, that all analyses were repeated using all possible pairs in the three genera with four species. Results were qualitatively identical. Each congeneric pair comprises one narrowly endemic species either restricted to the central and southern Pacific slope of Costa Rica, or, in some cases, reaching western Panama or the Caribbean slope in Costa Rica, and one species distributed more widely. The selection of endemic species was limited to tree genera that include regionally sympatric species with larger range sizes. For reasons of feasibility, our selection focused on species documented from known localities, in particular in case of the rarer endemic species (Table 1). Among possible widespread congeners, we selected those found growing in the neighbourhood of our sample of endemics (see below).

**Table 1:** The species used in the analysis, their classification as either widespread or endemic, their extent of occurrence (in km²) and the number of individuals sampled (N).

Family	Species name	Range size class	N	Extent of Occurrence
Annonaceae	*Guatteria amplifolia* Triana & Planch.	widespread	10	1.02 • 10^6^
Annonaceae	*Guatteria chiriquiensis* R. E. Fr.	endemics	9	8.60 • 10^3^
Annonaceae	*Guatteria pudica* N.Zamora & Maas	endemics	16	6.87 • 10^2^
Annonaceae	*Guatteria rostrata* Erkens & Maas	widespread	10	6.45 • 10^4^
Annonaceae	*Unonopsis osae* Maas & Westra	endemics	10	7.54 • 10^2^
Annonaceae	*Unonopsis theobromifolia* N. Zamora & Poveda	widespread	10	2.81 • 10^4^
Araliaceae	*Dendropanax arboreus* (L.) Decne. & Planch.	widespread	10	7.69 • 10^6^
Araliaceae	*Dendropanax ravenii* M. J. Cannon & Cannon	endemics	10	1.96 • 10^3^
Boraginaceae	*Cordia cymosa* (Donn. Sm.) Standl.	widespread	8	3.66 • 10^5^
Boraginaceae	*Cordia liesneri* J. S. Mill.	endemics	9	4.07 • 10^3^
Burseraceae	*Protium panamense* (Rose) I. M. Johnst.	widespread	8	1.98 • 10^5^
Burseraceae	*Protium pecuniosum* D. C. Daly	endemics	10	1.48 • 10^3^
Clusiaceae	*Chrysochlamys glauca* (Oerst. ex Planch. & Triana) Hemsl.	widespread	10	3.79 • 10^5^
Clusiaceae	*Chrysochlamys skutchii* Hammel	endemics	9	2.38 • 10^4^
Clusiaceae	*Garcinia aguilari* Hammel	endemics	10	9.43 • 10^1^
Clusiaceae	*Garcinia magnifolia* (Pittier) Hammel	widespread	10	1.64 • 10^5^
Euphorbiaceae	*Sapium allenii* Huft	endemics	11	8.89 • 10^2^
Euphorbiaceae	*Sapium glandulosum* (L.) Morong	widespread	10	1.35 • 10^7^
Fabaceae	*Inga skutchii* Standl.	endemics	10	7.79 • 10^3^
Fabaceae	*Inga spectabilis* (Vahl) Willd	widespread	9	2.51 • 10^6^
Lauraceae	*Ocotea mollifolia* Mez & Pittier	widespread	10	1.14 • 10^5^
Lauraceae	*Ocotea rivularis* Standl. & L. O. Williams	endemics	9	6.68 • 10^2^
Melastomataceae	*Miconia dissitinervia* Kriebel, Almeda & A. Estrada	endemics	11	3.75 • 10^3^
Melastomataceae	*Miconia donaeana* Naudin	widespread	10	1.22 • 10^6^
Melastomataceae	*Miconia osaensis* Aguilar, Kriebel & Almeda	endemics	10	9.61 • 10^1^
Melastomataceae	*Miconia trinervia* (Sw.) D. Don ex Loudon	widespread	10	5.89 • 10^6^
Primulaceae	*Ardisia compressa* Kunth	widespread	9	1.44 • 10^6^
Primulaceae	*Ardisia dunlapiana* P. H. Allen	endemics	10	1.44 • 10^3^
Rubiaceae	*Faramea occidentalis* (L.) A. Rich.	widespread	11	1.18 • 10^7^
Rubiaceae	*Faramea permagnifolia* Dwyer ex C. M. Taylor	endemics	12	5.38 • 10^1^
Sapotaceae	*Pouteria lecythidicarpa* P. E. Sánchez & Poveda	endemics	10	1.33 • 10^4^
Sapotaceae	*Pouteria subrotata* Cronquist	widespread	8	1.72 • 10^6^
Sapotaceae	*Pouteria torta* (Mart.) Radlk.	widespread	10	1.08 • 10^7^
Sapotaceae	*Pouteria triplarifolia* C. K. Allen ex T. D. Pennington	endemics	6	2.41 • 10^3^

### Field work

We collected samples during the rainy season 2015 (March to October). We tried to sample at least ten individuals per species. Some of the species were too rare, however, to accomplish a full sample (Table 1). We collected 82 individuals previously located in permanent plots and 253 trees outside of these plots. After sampling a tree of an endemic species, we tried to locate an individual of its widespread congener as close to it as possible, usually within a radius of 1000 m. We tried to avoid ontogenetic effects on trait variation by selecting only mature individuals (classified as such based on their diameter at breast height). A subsequent test confirmed that this sampling strategy had largely removed effects of tree size on trait values (S1 Fig). For each species, we sought individuals as spatially separated as possible to avoid sampling siblings. All sampled trees were growing within a 35 km radius.

We collected five fully expanded, mature leaves with no signs of damage and one wood core from each tree (S1 Text). For each leaf of each tree, we measured or calculated four functional traits: leaf area (LA), leaf dry matter content (LDMC), leaf thickness (LT), and specific leaf area (SLA) according to standard protocols [49]. For each tree, we additionally measured wood specific gravity (WSG) on a collected wood core. Details on measurement methods are provided in the supplementary material (S1 Text). On a pooled leaf sample per individual, we further measured leaf nitrogen content (N) and leaf phosphorus content (P) and calculated the leaf N:P ratios. Leaf N was measured by dry combustion using an autoanalyzer Rapid Exceed (Elementar, Langenselbold, Germany), and leaf P by acid digestion and inductively coupled plasma-optical emission spectroscopy (ICP-OES) using a spectrometer Optima 8300 (Perkin Elmer, Waltham, US) at the laboratory of the Agronomic Research Center (Centro de Investigaciones Agronómicas) of the University of Costa Rica.

### Environmental variation

To ease interpretation of possible differences in trait variation among congeneric species we also sampled a number of environmental covariates. For each tree, we measured the slope of the growing site (using a clinometer) and estimated crown exposure to light using an index from 0 to 5 [50]. Moreover, we took geographical coordinates using a GPS device (Garmin 60 CSX, mean RSE: 6 m). Based on these coordinates, we extracted the values from six not too closely correlated (Spearman's correlation coefficient < 0.7) bioclimatic variables from Worldclim (resolution ~1 km) (S1 Table) [40]. These variables were: annual mean temperature, mean diurnal temperature range, isothermality, (ratio of day-to-night temperature oscillation to summer-to-winter oscillation), annual precipitation, precipitation seasonality and precipitation of warmest quarter. We performed a PCA with those bioclimatic variables after normalization by means of z-scores. The scores of the first ordination axis, which explained the 86% of the variation was then used to characterize the mesoclimatic environment of each sampled tree individual. To obtain positive values for all trees, we added the absolute of the overall minimum value to all the PCA scores. From these values, we calculated the coefficient of variation of the climatic environment for each species. We also calculated the coefficient of variation for the slope of growing sites and the crown exposure to light.

### Functional variation and dispersion

Similar to the sampled environmental variables, we calculated the coefficient of variation (CV) for each trait, separately for each species. Because the species differed in sample size, we corrected the CV for unequal sample size assuming a normal distribution for each trait within the species [51]. A subsequent test confirmed that this correction had successfully removed possible bias from uneven sample sizes (S2 Table). To account for the variability of species in multidimensional trait space, we computed the functional dispersion of each species using the index proposed by Laliberté & Legendre [52]. This index is the average of the Euclidean distance between each individual and the centroid of all individuals per species in an ordination space. To calculate the index, first, the traits were scaled using standard scores and then subject to a principal component analysis to guarantee orthogonality. For the principal component analysis, we removed the LDMC and leaf N:P because these variables were calculated from other variables included in the PCA. We chose the first five principal components, which accounted for the 93% of the variance (S3 Table). We selected five components because this was the maximum the algorithm could use without a reduction of dimensionality [53]. Finally, we calculated the functional dispersion index using the package "FD" in R [53].

### Geographical range size

We defined a species’ geographical range size as the extent of occurrence (EOO) *sensu* Gaston & Fuller [4]. For each species, we collected geographic coordinates of occurrences from different sources through the Global Biodiversity Information Facility (GBIF) (S4 Table) and own field records during the collection of samples. We removed or checked the following kind of occurrences: a) uncertain occurrences, i.e. those separated from the nearest other record at least twice the mean distance between all records and with locality descriptions that suggest that species were planted in parks or gardens, b) duplicated occurrences inside of the same 1x1 km cell in a raster map, and c) occurrences without detailed information about locality. We constructed a polygon based on an α-hull around the occurrence localities [54] using the R package “alphahull” [55]. For each species, we constructed the α-hull using 8 as α value because it was the smallest value to obtain polygons with all internal angles greater than 0 that included all the occurrence points of the respective species. The EOO was then calculated from the intersection of the α-hull and the continental contour map (projected by a Lambert Equal Area Projection).

### Statistical analysis

For a more detailed description of trait variability, we decomposed the variance of each functional trait across three scales: genera, species, trees. We used the method described by Messier et al. [14][14] which fits a generalised linear model to the hierarchically nested variances (across scales). The trait values were normalized using log transformations. For the model and variance decomposition, we used the R-packages "nlme" [56] and "ape" [57].

We used two alternative analytical approaches to compare the intraspecific variation of traits between endemic and widespread species. First, the CV of each trait was compared between widespread and endemic species by testing whether the differences among congeneric species pairs significantly differ from zero, on average. We, therefore, used a linear mixed effects model with this difference as response and the intercept as the only term on the right-hand side of the model equation. To account for the phylogenetic structure in the data, we additionally estimated a random intercept for each genus in the mixed model. We used the same model structure to compare the CV of the environmental variables (crown exposure to light, slope of the growing sites and climate) between congeneric species with contrasting range size to consider that effect in the interpretation of the results. Moreover, we tested the correlation between the magnitude of trait values and environmental variables by means of linear mixed effects models with a random effect for species identity. Finally, we also tested whether similarity of trait values among individuals depends on geographical distances between them using Mantel tests, separately for each trait and species.

In a second analysis, we tested whether the CV of individual traits could successfully predict the species’ range size. We, therefore, used a linear mixed effects model with the log-transformed extent of occurrence as the response, the CV as the predictor and the genus as a random factor. Finally, we applied both approaches to the multivariate trait space, i.e. we (1) compared functional dispersion indices between the 17 pairs of endemic and widespread species and tested whether the average difference among congeneric species pairs significantly differed from zero; and (2), we tested whether the functional dispersion could predict the log-transformed range size. We used likelihood ratio tests to assess the statistical significance of regressions terms.

To back-up our results, we additionally ran an analysis that included variation of environmental variables at the tree level directly. We, therefore, first, regressed trait values of individual trees against each environmental variable (climate, crown light exposure, slope inclination), separately for each trait, species and environmental variable. Environmental variables predicting trait values in these uni-variable regression models (p-value < 0.1) were combined in one linear model per species and trait (S2 Fig). We then retained those environmental variables with a p-value < 0.05 in these (potential) multiple regression models. From these final models, we extracted the residuals and added the original trait mean to each residual to preserve the original measurement scale. The resulting values were used in subsequent analyses. They represent the individuals’ variability in the respective trait that could not be explained by the measured dimensions of the physical environment, and in cases where species traits were uncorrelated to any environmental variable, the original trait values were retained. With these new values, we estimated, again, the CV for each trait and the functional dispersion index across all the traits and compared these metrics between widespread and endemic species using the same procedures as described above.We ran all analyses in R 3.3.1 [58].

## Results

The range size of our study species ranged from 5.37·10^1^ to 2.38 ·10^4^ km^2^ for endemic species and from 2.80·10^4^ to 1.34·10^7^ km^2^ for widespread species (Table 1, S1 File). The smallest difference between species in a congeneric pair (endemic-widespread) was 2.7·10^4^ km^2^ between the two species in the genus *Unonopsis* (Table 1), while the ratio between the maximum and minimum range size was between 7.5, in one of the pairs in the genus *Guatteria*, and 2.18·10^5^ in the genus *Faramea* (Table 1, S1 File).

**Fig 2:**
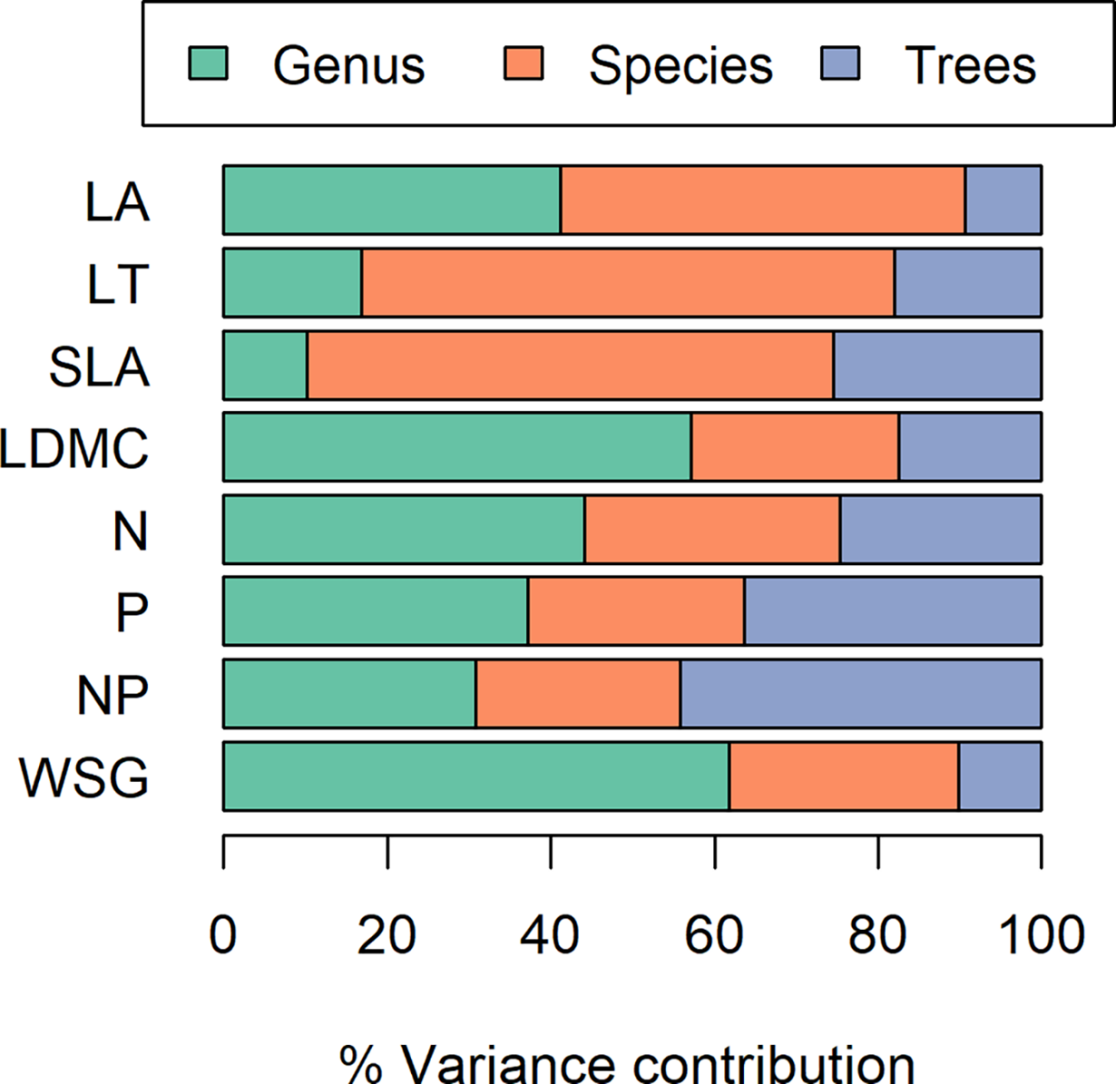
Partitioning of the nested variance in eight functional traits measured in 34 tropical tree species. Leaf area (LA), leaf thickness (LT), specific leaf area (SLA) and leaf dry matter content (LDMC), wood specific gravity (WSG), leaf nitrogen content (N) and leaf phosphorus content (P), and N:P ratio (NP).

We sampled 335 individual trees of the selected 34 species (Table 1). Among the species analysed, functional traits varied with respect to the magnitude of CV and in how the variance was partitioned among levels of biological organization (Fig 2, S5 Table). For traits such as WSG and LDMC, CVs were low (averages ± 1 standard deviation (SD): 0.09 ± 0.05 and 0.09 ± 0.04, respectively (S3 Fig) and most variance was explained by differences between genera (61.8% and 57.1% respectively, Fig 2). For other traits like LA and SLA, CVs were much higher (averages ± 1 SD: 0.28 ± 0.11 and 0.16 ± 0.07 respectively, S3 Fig) and most of the variance was explained by differences between species (49.4% and 64.4%, respectively, Fig 2). The part of total trait variance explained by variation within trees ranged from c. 9.31% for LA to about 45% in NP (Fig 2).

The CVs of the three environmental variables did not differ between congeneric species with contrasting range size (S4 Fig). Concerning the magnitude of trait values, the climatic environment had an effect on LT and WSG. LDMC and LT increased, and SLA decreased with crown light exposure. Slope inclination was not related to the value of any trait (S6 Table). Mantel tests demonstrate that there is limited correlation between similarity of trait values and geographical distance among species (32 significant correlations out of 272, S5 Fig). Local (genetic) adaptation of trait values hence seems to play a relatively minor role within the regional populations of both widespread and endemic species.

**Fig 3:**
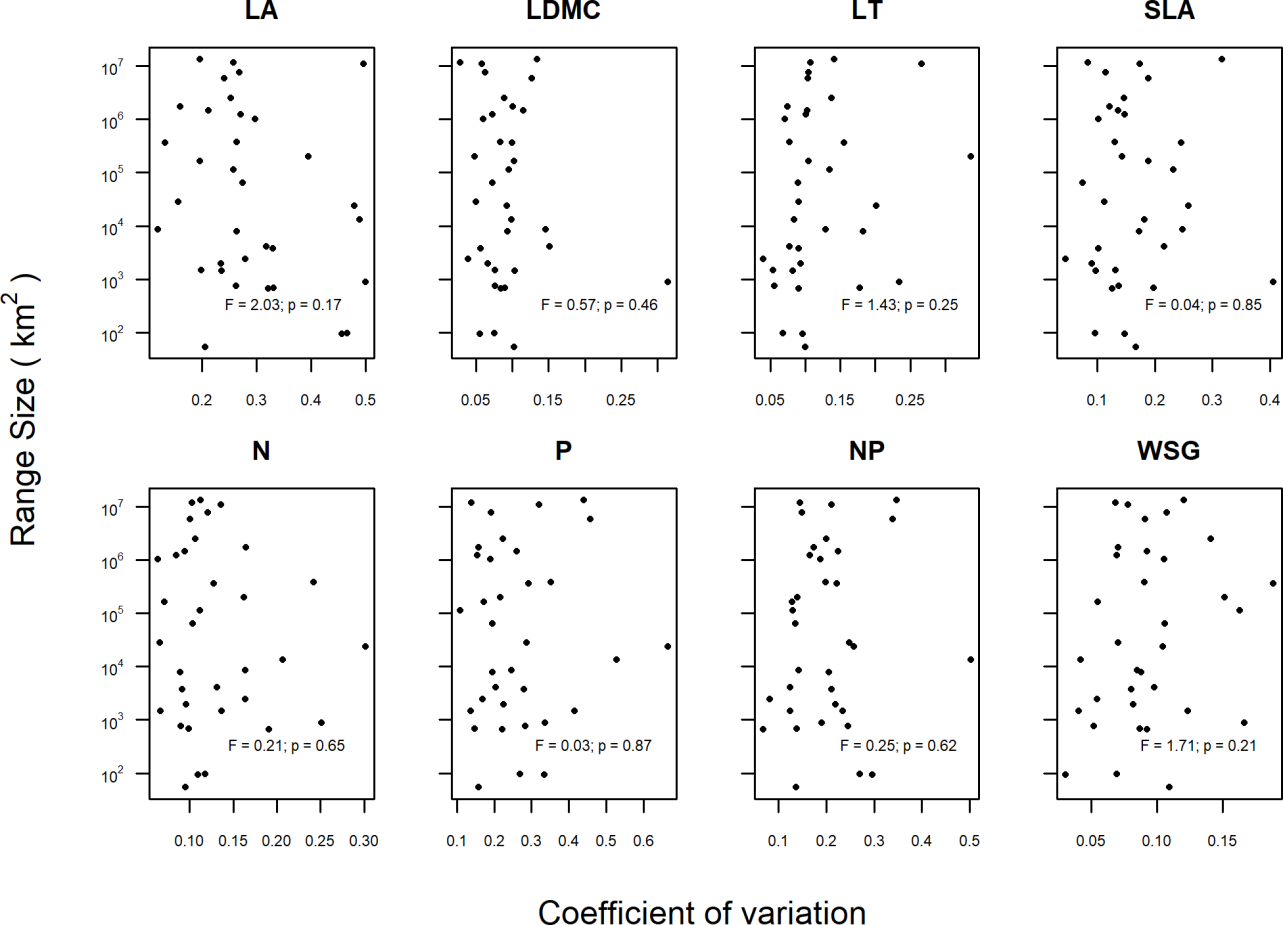
Coefficients of variation (CV) in eight functional traits printed against range size of 34 tropical tree species. Functional traits: leaf area (LA), leaf dry matter content (LDMC), leaf thickness (LT), specific leaf area (SLA), leaf nitrogen content (N), leaf phosphorus content (P), N:P ratio (NP) and wood specific gravity (WSG). The test represents the F-value and the correspondent p-value of a generalised linear mixed effects model testing the dependence of range size on CV and using genus as a random factor. All F-tests used 1 and 19 numerator and denominator degrees of freedom, respectively.

**Fig 4:**
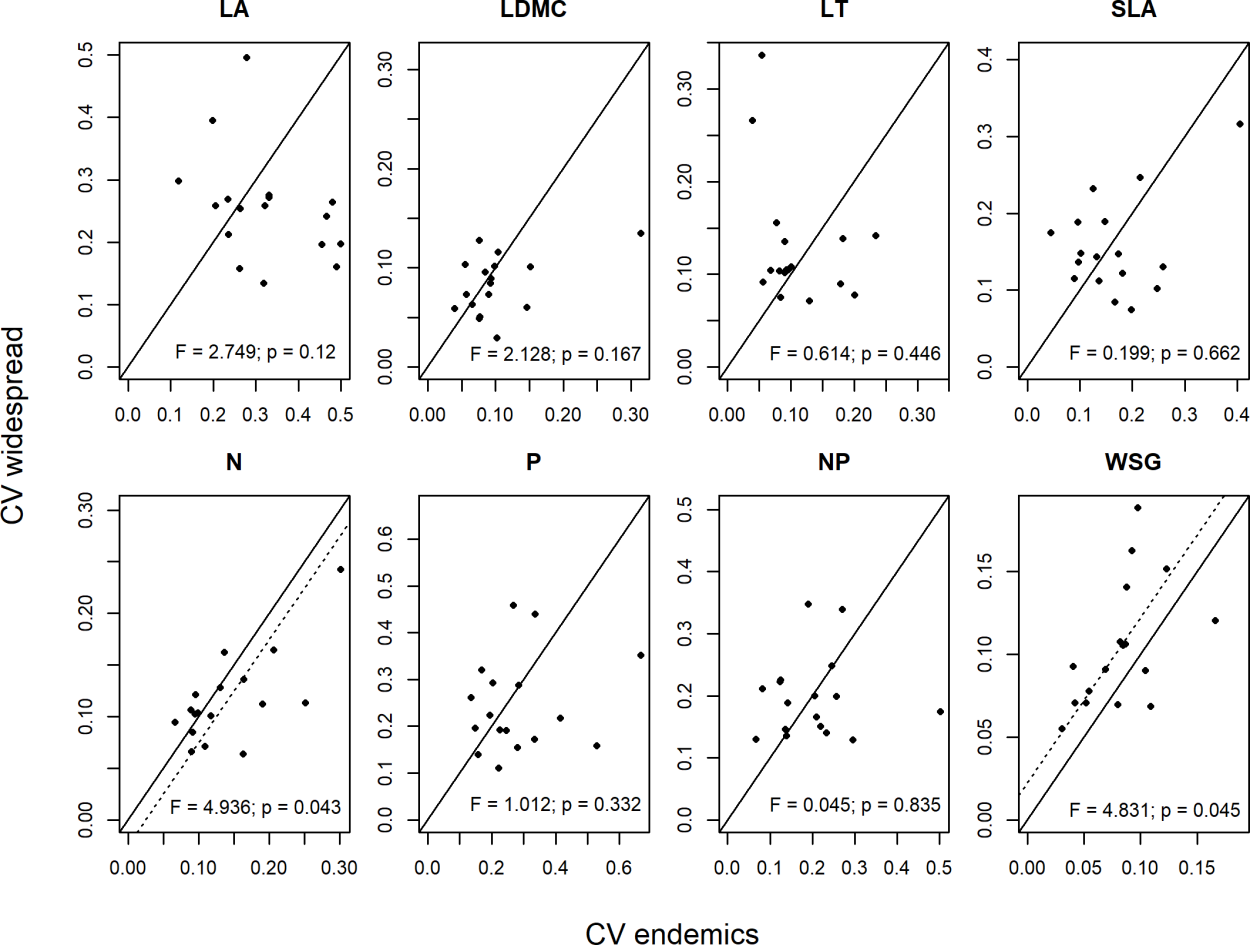
Coefficients of variation (CV) in eight functional traits of 17 congeneric pairs of endemic neotropical tree species and their widespread congeners. Functional traits: leaf area (LA), leaf dry matter content (LDMC), leaf thickness (LT), specific leaf area (SLA), leaf nitrogen content (N),leaf phosphorus content (P), N:P ratio (NP), and wood specific gravity (WSG). Each point represents one pair (endemic, widespread). The continuous diagonal represents the null model, i.e. positioning of points along the line indicates equal trait variability of both species in a pair. Points above the line represent pairs with CV higher in widespread species, and points below the line pairs with CV higher in endemic species. The dotted diagonal represents the mean difference between pairs in case this difference was statistically significant.

**Fig 5:**
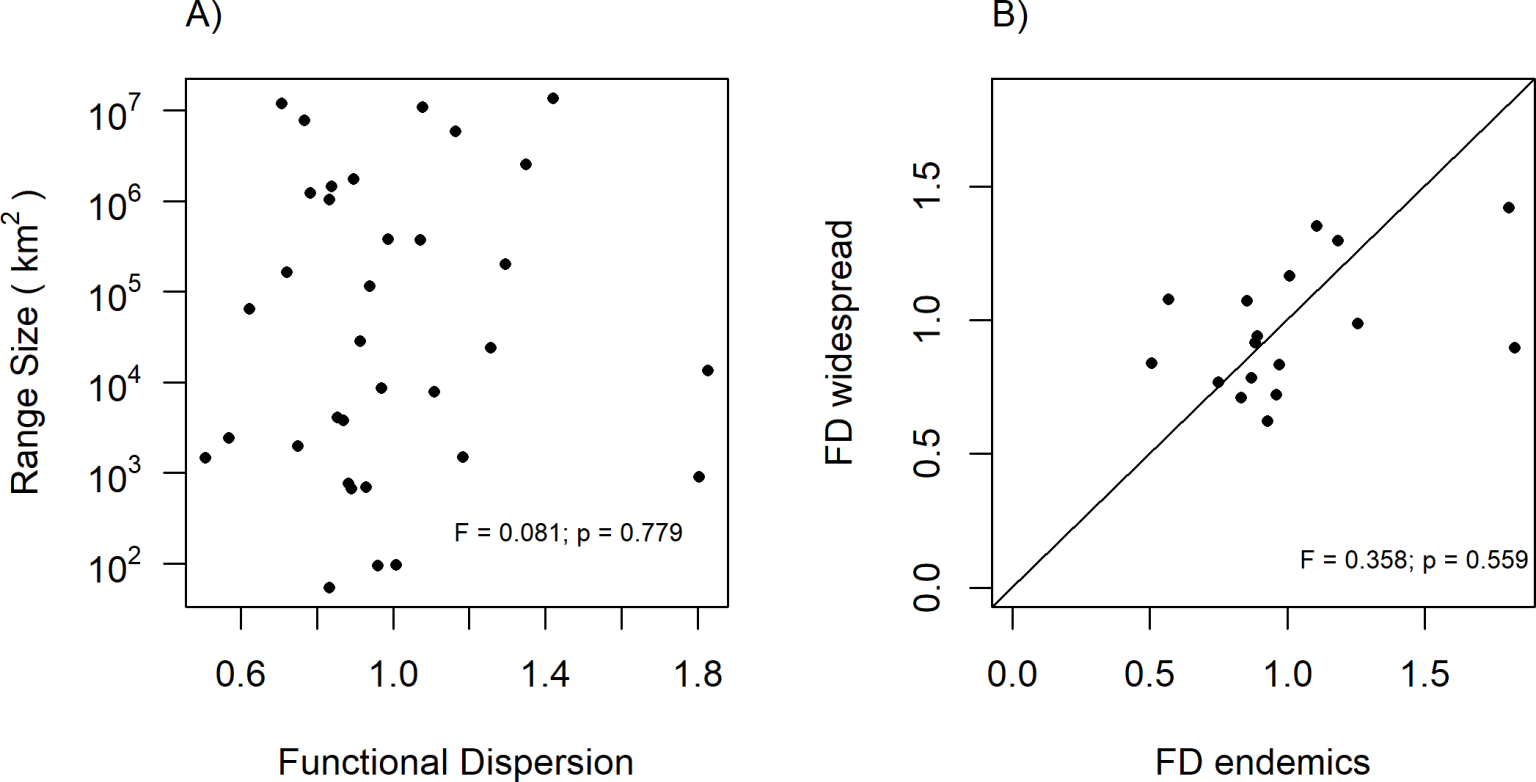
Functional dispersion, calculated from six functional traits of the studied 34 tropical tree species. A) Functional dispersion in relation to the range size. B) Functional dispersion (FD) of 17 congeneric pairs of endemic species and their widespread congeners. In Fig 5B each point represents one pair (endemic, widespread). The diagonal represents the null model, i.e. positioning of points along the line indicates equal functional dispersion of both species in a pair. Points above the line represent pairs with FD higher in widespread species, and points below the line pairs with FD higher in endemic species.

The CV of none of the traits could significantly explain species’ range sizes (Fig 3). Similarly, endemic and widespread congeners did hardly differ in trait CVs, even if variation in WSG was marginally significantly higher, and variation in leaf N marginally significantly lower in widespread species (Fig 4, S6 Fig). The functional dispersion index, as a multivariate metric, could neither explain species’ range sizes (Fig 5) nor was it different between endemic and widespread species (Fig 5, S7 Fig).

The alternative analysis using environmental variables as covariates provided qualitatively identical results. The CV of the functional traits was not different between endemic and widespread species (S8 Fig). Overall, there was no single trait for which trait variability was significantly larger in the widespread species. The functional dispersion neither explained the EOO (Figure A in S9 Fig) nor did it differ between the two groups (Figure B in S9 Fig).

## Discussion

Trait variability among individuals of the same species makes an important contribution to community-level trait variation in general [24]. Although our results demonstrate that for the traits considered interspecific and intergeneric trait differences predominate, the scale of intra-specific variability (10–45%) is similar to the global average of 25% found in the recent meta-analysis [24]. For tree species in the tropical forest of Panama, near to our own study area, this intra-specific trait variation has been demonstrated to compensate for species turnover among local plots of similar environments reducing trait differences among these plots to a low level [14]. Taken together, these results indicate that the local environment exerts a filter on the traits of individuals, at least in neotropical forests of Central America. As a corollary, species with larger intra-specific trait variability should indeed be able to occur at more diverse environments and thus, eventually, occupy larger ranges across the neotropical forest biome as environmental variation tends to increase with spatial scale [26]. Nevertheless, our results did not provide support for a relationship between the local intra-specific trait variation and range size in the sampled tree genera. Trait variability, measured separately for individual traits or as a combined metric across several traits, does not predict range sizes of the 34 tree species considered nor does it differ substantially among the 17 pairs of widespread and endemic congeners. Several reasons may be responsible for these findings.

First, species may differ in how an individual trait responds to the same ecological gradient. If trait-environment relationships vary among species, e.g. if the same difference in wood specific gravity results in a different decrease of drought-induced mortality [38], different levels of trait variation are necessary for the two species to cope with the same variation in environmental conditions. Vice versa, the same level of trait variability allows for coping with different levels of environmental heterogeneity, i.e. it results in distinct niche breadth and hence, potentially, also range size. Indeed, differences in the slope of trait-environment correlations among species have repeatedly been reported [59] and may result from processes of general phenotype integration [60]. Moreover, the effect of trait variability on niche breadth may not be independent of the trait mean, i.e. the same amount of variability may convey higher environmental tolerance under lower or higher average trait values. For instance, species with high xylem hydraulic vulnerability are found in high and low rainfall regions, but species with low vulnerability are rare in regions with high rainfall [61]; similar patterns can be described with leaf size, with small leaves found in high and low rainfall regions, but species with large leaves being rare in regions with low rainfall [62]. Finally, several environmental variables often simultaneously affect many, partly interdependent traits [59,62]. These interactions may result in compensation effects, with species maintaining high fitness levels along an environmental gradient despite little variation in a particular trait but variation in other traits [63].

Second, maximum or average trait values of species may be more important for geographical success than trait variability, as has been shown for some groups of trees with respect to e.g. maximum height, WSG and N [64,65]. The importance of absolute trait values may result, for example, from the competitive superiority they provide [37]. From the traits analyzed here, leaf nitrogen content is, for example, linked directly to photosynthetic rate [66], which in turn is related to growth and demographic processes of survival and recruitment [67], and hence also to competitive ability [37,68]. If the competitive ability is more important in determining species’ rate of geographical expansion and eventual range size [69,70] than environmental tolerance, then absolute values of these traits may be more closely linked to range sizes than trait variation, and selection may generally disfavour variability of these traits in successful species. More generally, the idea of a causal link between biogeographical success and intra-specific trait variation may overlook the possible negative effects of inherently high trait variability [63,71]. Indeed, too large variation can be maladaptive, especially on a local scale where correlated environmental conditions exert selective pressures on populations towards phenotypic stability [63]. The balance between negative and positive effects of trait variability may depend on the harshness of environmental conditions, i.e. the strength of environmental filtering in a species preferred habitat [72,73]. In line with this idea, our data actually indicate that species with higher WSG, i.e. those likely adapted to drought [38], had proportionally lower variation in this trait than species with low WSG (S10 Fig). We moreover emphasize that further evaluations of the correlation between trait variability and range size should include aspects of evolutionary history and clade age [74], and account for differential evolutionary constraints on the variability of individual traits. Here, we tried to minimize confounding effects of evolutionary history by focusing on congeneric species pairs. However, even the individual species in these pairs may substantially differ in evolutionary age and these differences may have had an important impact on current range sizes. Unfortunately, detailed phylogenies are currently available for only three of the studied genera (*Dendropanax*, *Guatteria* and *Protium*) [75–77]. From the coarse information deducible from these phylogenies, effects of evolutionary age on range size differences are not apparent (S7 Table), but additional data for other genera, and with a higher temporal resolution, may change these conclusions.

Third, documented intra-specific trait variation across the entire geographical distribution of a species, including that of traits analyzed here [23,78,79] may actually be a result of range expansion rather than a prerequisite. Indeed, our data do not provide any support for the idea that inherent trait variability begets large range sizes [6]. However, they do not, exclude that large range sizes beget high trait variability at the whole-range scale.

Fourth, range size is of course not exclusively controlled by the traits studied here. For example, traits related to the reproduction, dispersal and migration of species, such as preferred dispersal vector, seed size, or mating system, are likely important for range expansion [74,80,81]. The information available for the species studied here is not sufficient for a quantitative analysis of these effects. However, the available literature data do not suggest that seed traits or predominant dispersers differ saliently between congeneric widespread and endemic species pairs, nor did we find any evidence for their impact on range sizes in our data (S8 Table). In fact, within-genus variation is often low for these two traits [82,83]. As a corollary, while these traits certainly affect biogeography [80,81], they are unlikely to have a major effect on range differences among closely related species

Finally, we emphasize that our results do not strictly falsify intra-specific variability as a driver of range size [84]. In particular, our regional-scale study may not have captured the full extent of inherent heritable trait variability, particularly in widespread species. However, as the sampling sites did vary in environmental conditions at least to a certain extent, our data suggest that the observed level of environmental variation did not trigger the display of larger trait variability in species with larger ranges. This finding suggests that these species are not per se more flexible when confronted with varying environments.

## Supporting information

S1 Text Methods of functional trait measurements. (DOCX)

S1 Table Correlation between bioclimatic variables within the tropical region (23.5° N-23.5°S) in America. (XLSX)

S2 Table Coefficients estimated (β) ± 1 standard error and the associated test statistics for mixed effects models evaluating the effect of the sample size on the coefficient of variation for eight functional traits analysed in 34 neotropical trees species. (DOCX)

S3 Table Principal component analysis of six functional traits measured in 335 individual trees of 34 species. The functional traits included in the analysis were: Leaf area (LA), leaf thickness (LT), specific leaf area (SLA), leaf nitrogen content (N), leaf phosphorus content (P), and wood specific gravity (WSG).
(DOCX)

S4 Table Global Biodiversity Information Facility Data Providers. (XLSX)

S5 Table Coefficients of variation of eight functional traits and multivariate functional dispersion (FD) for 34 neotropical tree species. Traits: Leaf area (LA), leaf dry matter content (LDMC), leaf thickness (LT), specific leaf area (SLA), leaf nitrogen content (N), leaf phosphorus content (P), leaf N:P ratio (NP) and wood specific gravity (WSG).
(DOCX)

S6 Table Fixed effects coefficients (β ± 1 standard error), derived from linear mixed effects models, for the effects of environmental variables measured on eight functional traits in 335 individual trees of 34 species, using species identity as a random effect. The functional traits included in the analysis were: Leaf area (LA), leaf thickness (LT), specific leaf area (SLA), leaf dry matter content (LDMC), leaf nitrogen content (N), leaf phosphorus content (P), leaf nitrogen phosphorus ratio (NP) and wood specific gravity (WSG). Statistically significant results (Ho: β = 0) are in bold.
(DOCX)

S7 Table Estimated ages of species included in the present analysis according to available phylogenies [75–77]. (DOCX)

S8 Table Seed size (length x width) and seed dispersers of the 34 neotropical tree species used in the analysis. B: Birds, M: mammals.
(DOCX)

S1 Fig Pearson's correlation coefficients between tree size (diameter at breast height) and eight functional traits in 34 neotropical tree species. Correlation coefficients significantly different from zero (p<0.05, 20 out of 272) are presented with color.
(TIFF)

S2 Fig Graphical display of the univariate linear regression models of eight functional traits against three environmental variables (climate, crown light exposure and slope inclination of growing site). Red squares indicate models with p-value< 0.1. Functional traits are abbreviated as follows: leaf area (LA), leaf dry matter content (LDMC), leaf thickness (LT), specific leaf area (SLA), leaf nitrogen content (N), leaf phosphorus content (P), leaf nitrogen to phosphorus ratio (N:P) and wood specific gravity (WSG). Species codes are in S5 Table.
(TIFF)

S3 Fig Frequency distributions of the coefficients of variation of eight functional traits in 34 tropical trees species. The dotted vertical line indicates the mean of the coefficients of variation. Leaf area (LA), leaf dry matter content (LDMC), leaf thickness (LT), specific leaf area (SLA), leaf nitrogen content (N), leaf phosphorus content (P), leaf N:P ratio (NP) and wood specific gravity (WSG).
(TIFF)

S4 Fig Coefficients of variation (CV) of three environmental variables used to characterize the sampled trees’ growing sites: crown light exposure, the slope of the growing sites and local climate. Each point represents one pair of congeneric endemic and widespread species. The diagonal represents the null model, i.e. positioning of points along the line indicates equal environmental variability among the sampled trees of both species in a pair. Points above the line represent pairs with environmental CV higher in widespread species and points below the line pairs with CV higher in endemic species.
(TIFF)

S5 Fig Mantel tests (based on Pearson's correlation coefficient) between the geographical distance of individuals and the absolute difference between the trait values of the individuals for eight functional traits in 34 neotropical tree species. Correlation coefficients significantly different from zero (p<0.05) are presented with color.
(TIFF)

S6 Fig Coefficients of variation (CV) in eight functional traits using all combinations of possible pairs of endemic and widespread congeners from 17 genera of neotropical tree species. (TIFF)

S7 Fig Functional dispersion (FD) of 17 congeneric pairs of endemic neotropical tree species and their widespread congeners, using all combinations of possible pairs of endemic and widespread congeners from 17 genera of neotropical tree species. (TIFF)

S8 Fig Coefficients of variation (CV) in eight functional traits of 17 congeneric pairs of endemic neotropical tree species and their widespread congeners after removing variation attributable to three measured environmental variables. Leaf area (LA), leaf thickness (LT), specific leaf area (SLA) and leaf dry matter content (LDMC), wood specific gravity (WSG), leaf nitrogen content (N) and leaf phosphorus content (P), and N:P ratio of endemic neotropical tree species and their widespread congeners after removing variation related to variation in environmental variables. Each point represents one congeneric pair of species (endemic, widespread). The diagonal represents the null model, i.e. positioning of points along the line indicates equal trait variability of both species in a pair. Points above the line represent pairs with CV greater in widespread species, and points below the line pairs with CV greater in endemic species. The p-value is for the associated statistics testing if the intercept = 0.
(TIFF)

S9 Fig Functional dispersion calculated from six functional traits after removing variation attributable to variation in measured environmental variables for the studied 34 tropical tree species. A) Functional dispersion in relation to the range size. B) Functional dispersion (FD) of 17 congeneric pairs of endemic species and their widespread congeners. In Fig 5B each point represents one pair (endemic, widespread). The diagonal represents the null model, i.e. positioning of points along the line indicates equal functional dispersion of both species in a pair. Points above the line represent pairs with FD higher in widespread species, and points below the line pairs with FD higher in endemic species.
(TIFF)

S10 Fig Linear relationships between the mean and the coefficient of variation of wood specific gravity and leaf nitrogen content among the 34 neotropical tree species analyzed. The regression line is represented by a solid line when the effect of the regressor was significantly different from zero, and with a dotted line when it was not significant.
(TIFF)

S1 File Maps of the geographic ranges of the 34 tree species studied. (DOCX)
