# Supplementary material for: Is local trait variation related to total range size of tropical trees?
Source: PLoS One. 2018 Mar 7;13(3):e0193268. doi: 10.1371/journal.pone.0193268 (PMC5841763; doi:10.1371/journal.pone.0193268)
Supplement: S1 Text — (DOCX) [file pone.0193268.s001.docx]

**S1 Text. Methods of functional trait measurements**

Leaf thickness was measured using a Mitutoyo 7321 dial thickness gauge (readability 0.01 mm) (Mitutoyo American Corp.). We took three measurements, at the proximal, medium and distal part of the leaf lamina, avoiding major veins. The values used are an average of the three measurements. Leaf fresh weight and dry weight after drying 72 hours at 70 ºC were measured using a Radwag WTB 200 Precision Balance (readability 1 mg) (Radwag). The leaves of species with compound leaves (genera *Inga* and *Protium*) were weighed with the petiole because it is hard to separate from the rachis; the leaves within a genus were always either compound or simple but never both. Leaf area was measured using a LICOR-3100 area meter (LI-COR Inc.); the values used are an average of three measurements. SLA was calculated as leaf fresh area measured in cm^2^ divided by leaf dry weight measured in mg. LDMC was estimated as leaf dry weight measured in mg divided by leaf fresh weight measured in g.

Wood specific gravity (WSG) was estimated as dry weight divided by the fresh volume of each wood core. The volume was measured using the Archimedes principle. After drying for 72 hours at 103 ºC, wood samples were weighted on a precision balance (to 1 mg). We corrected measures using the value of water density at 26 ºC, which was the average temperature in the laboratory; therefore the values are expressed as WSG without units.
