# Supplementary material for: Is local trait variation related to total range size of tropical trees?
Source: PLoS One. 2018 Mar 7;13(3):e0193268. doi: 10.1371/journal.pone.0193268 (PMC5841763; doi:10.1371/journal.pone.0193268)
Supplement: S2 Table — (DOCX) [file pone.0193268.s003.docx]

S2 Table. Coefficients estimated (β) ± 1 standard error and the associated test statistics for the mixed effects models evaluating the effect of the sample size on the coefficient of variation for eight functional traits analyzed in 34 tropical trees species. Traits: Leaf area, LA; leaf thickness, LT; specific leaf area, SLA; leaf dry matter content, LDMC; leaf nitrogen content, N; leaf phosphorus content, P; leaf nitrogen-phosphorus ratio, NP; and wood specific gravity, WSG. The species were used as a random factor in each model.

| Trait | β | Std. Error | DF | t. value | p. value |
| --- | --- | --- | --- | --- | --- |
| LA | 0.003 | 0.0022 | 198 | 1.386 | 0.167 |
| LT | -0.0005 | 0.0007 | 198 | -0.71 | 0.476 |
| SLA | -0.0011 | 0.0009 | 198 | -1.13 | 0.259 |
| LDMC | 0.0001 | 0.0006 | 198 | 0.228 | 0.819 |
| N | 0.0012 | 0.0011 | 198 | 1.108 | 0.269 |
| P | 0.0022 | 0.0018 | 198 | 1.261 | 0.208 |
| NP | 0.0019 | 0.0012 | 198 | 1.618 | 0.107 |
| WSG | 0.0001 | 0.0006 | 198 | 0.103 | 0.917 |

Method: For each trait and each species, we calculated the coefficient of variation (CV) for subsamples of incremental size, starting with n=4 until n=all samples taken (10, in most cases). Subsamples were randomly selected. For each trait, we subsequently fitted a linear mixed effects model of the effect of the sample size on CV, using species identity as a random factor. Estimated coefficents are the fixed effect estimates of this linear mixed effects model.
