# Supplementary material for: Is local trait variation related to total range size of tropical trees?
Source: PLoS One. 2018 Mar 7;13(3):e0193268. doi: 10.1371/journal.pone.0193268 (PMC5841763; doi:10.1371/journal.pone.0193268)
Supplement: S3 Table — The functional traits included in the analysis were: Leaf area (LA), leaf thickness (LT), specific leaf area (SLA), leaf nitrogen content (N), leaf phosphorus content (P), and wood specific gravity (WSG). (DOCX) [file pone.0193268.s004.docx]

S3 Table. **Principal component analysis of six functional traits measured in 335 individual trees of 34 species.** The functional traits included in the analysis were: Leaf area (LA), leaf thickness (LT), specific leaf area (SLA), leaf nitrogen content (N), leaf phosphorus content (P), and wood specific gravity (WSG).

|  | PC 1 | PC 2 | PC 3 | PC 4 | PC 5 |
| --- | --- | --- | --- | --- | --- |
| Variance | 2.699 | 1.514 | 1.032 | 0.739 | 0.55 |
| % of variance | 38.554 | 21.622 | 14.736 | 10.554 | 7.86 |
| Cumulative % of Variance | 38.554 | 60.177 | 74.913 | 85.467 | 93.327 |
