# Supplementary material for: Is local trait variation related to total range size of tropical trees?
Source: PLoS One. 2018 Mar 7;13(3):e0193268. doi: 10.1371/journal.pone.0193268 (PMC5841763; doi:10.1371/journal.pone.0193268)
Supplement: S5 Table — Traits: Leaf area (LA), leaf dry matter content (LDMC), leaf thickness (LT), specific leaf area (SLA), leaf nitrogen content (N), leaf phosphorus content (P), leaf N:P ratio (NP) and wood specific gravity (WSG). (DOCX) [file pone.0193268.s006.docx]

S5 Table. Coefficients of variation of eight functional traits and multivariate functional dispersion (FD) for 34 neotropical tree species. Traits: Leaf area (LA), leaf dry matter content (LDMC), leaf thickness (LT), specific leaf area (SLA), leaf nitrogen content (N), leaf phosphorus content (P), leaf N:P ratio (NP) and wood specific gravity (WSG).

| Species Code | LA | LDMC | LT | SLA | N | NP | P | WSG | FD |
| --- | --- | --- | --- | --- | --- | --- | --- | --- | --- |
| ARCOM | 0.212 | 0.115 | 0.103 | 0.136 | 0.095 | 0.225 | 0.260 | 0.092 | 0.839 |
| ARDUN | 0.237 | 0.104 | 0.082 | 0.097 | 0.067 | 0.125 | 0.137 | 0.041 | 0.507 |
| CHGLA | 0.264 | 0.084 | 0.078 | 0.130 | 0.242 | 0.198 | 0.352 | 0.090 | 0.986 |
| CHSKU | 0.480 | 0.092 | 0.202 | 0.259 | 0.302 | 0.257 | 0.666 | 0.104 | 1.257 |
| COCYM | 0.133 | 0.100 | 0.156 | 0.246 | 0.128 | 0.223 | 0.292 | 0.188 | 1.072 |
| COLIE | 0.319 | 0.151 | 0.077 | 0.216 | 0.131 | 0.124 | 0.204 | 0.098 | 0.854 |
| DEARB | 0.268 | 0.063 | 0.105 | 0.115 | 0.121 | 0.150 | 0.191 | 0.108 | 0.767 |
| DERAV | 0.235 | 0.066 | 0.094 | 0.090 | 0.096 | 0.220 | 0.225 | 0.082 | 0.750 |
| FAOCC | 0.258 | 0.029 | 0.108 | 0.084 | 0.103 | 0.146 | 0.139 | 0.068 | 0.708 |
| FAPER | 0.206 | 0.103 | 0.100 | 0.167 | 0.095 | 0.136 | 0.158 | 0.109 | 0.833 |
| GAAGU | 0.456 | 0.056 | 0.096 | 0.148 | 0.110 | 0.296 | 0.334 | 0.031 | 0.960 |
| GAMAG | 0.196 | 0.103 | 0.105 | 0.189 | 0.071 | 0.128 | 0.172 | 0.055 | 0.721 |
| GUAMP | 0.298 | 0.060 | 0.071 | 0.102 | 0.063 | 0.188 | 0.190 | 0.105 | 0.832 |
| GUCHI | 0.120 | 0.146 | 0.129 | 0.248 | 0.164 | 0.142 | 0.246 | 0.085 | 0.970 |
| GUPUD | 0.332 | 0.090 | 0.178 | 0.198 | 0.099 | 0.139 | 0.149 | 0.087 | 0.929 |
| GUROS | 0.275 | 0.073 | 0.089 | 0.074 | 0.103 | 0.135 | 0.195 | 0.106 | 0.623 |
| INSKU | 0.264 | 0.094 | 0.183 | 0.173 | 0.089 | 0.206 | 0.195 | 0.088 | 1.108 |
| INSPE | 0.254 | 0.089 | 0.138 | 0.147 | 0.106 | 0.200 | 0.224 | 0.141 | 1.350 |
| MIDIS | 0.331 | 0.057 | 0.090 | 0.102 | 0.092 | 0.211 | 0.281 | 0.080 | 0.869 |
| MIDON | 0.271 | 0.073 | 0.102 | 0.148 | 0.085 | 0.166 | 0.154 | 0.070 | 0.784 |
| MIOSA | 0.467 | 0.076 | 0.069 | 0.096 | 0.118 | 0.271 | 0.269 | 0.069 | 1.008 |
| MITRI | 0.241 | 0.127 | 0.104 | 0.189 | 0.101 | 0.339 | 0.459 | 0.091 | 1.163 |
| OCMOL | 0.258 | 0.095 | 0.135 | 0.232 | 0.112 | 0.130 | 0.110 | 0.163 | 0.938 |
| OCRIV | 0.322 | 0.085 | 0.090 | 0.126 | 0.191 | 0.068 | 0.222 | 0.092 | 0.891 |
| POLEC | 0.489 | 0.099 | 0.084 | 0.181 | 0.207 | 0.502 | 0.528 | 0.042 | 1.828 |
| POSUB | 0.160 | 0.101 | 0.075 | 0.122 | 0.164 | 0.174 | 0.158 | 0.070 | 0.896 |
| POTOR | 0.496 | 0.059 | 0.266 | 0.174 | 0.136 | 0.211 | 0.320 | 0.078 | 1.078 |
| POTRI | 0.280 | 0.040 | 0.040 | 0.045 | 0.164 | 0.082 | 0.170 | 0.054 | 0.568 |
| PRPAN | 0.395 | 0.048 | 0.337 | 0.143 | 0.162 | 0.140 | 0.216 | 0.151 | 1.295 |
| PRPEC | 0.200 | 0.077 | 0.054 | 0.132 | 0.137 | 0.234 | 0.415 | 0.123 | 1.184 |
| SAALL | 0.500 | 0.314 | 0.234 | 0.406 | 0.251 | 0.191 | 0.337 | 0.166 | 1.805 |
| SAGLA | 0.197 | 0.135 | 0.141 | 0.316 | 0.113 | 0.348 | 0.439 | 0.120 | 1.420 |
| UNOSA | 0.263 | 0.077 | 0.056 | 0.137 | 0.090 | 0.246 | 0.284 | 0.052 | 0.884 |
| UNTHE | 0.157 | 0.050 | 0.091 | 0.112 | 0.066 | 0.248 | 0.287 | 0.071 | 0.914 |

Species codes: *Ardisia* *compressa* (ARCOM), *Ardisia* *dunlapiana* (ARDUN), *Chrysochlamys* *glauca* (CHGLA), *Chrysochlamys* *skutchii* (CHSKU), *Cordia* *cymosa* (COCYM), *Cordia* *liesneri* (COLIE), *Dendropanax* *arboreus* (DEARB), *Dendropanax* *ravenii* (DERAV), *Faramea* *occidentalis* (FAOCC), *Faramea* *permagnifolia* (FAPER), *Garcinia* *aguilarii* (GAAGU), *Garcinia* *magnifolia* (GAMAG), *Guatteria* *amplifolia* (GUAMP), *Guatteria* *chiriquiensis* (GUCHI), *Guatteria* *pudica* (GUPUD), *Guatteria* *rostrata* (GUROS), *Inga* *skutchii* (INSKU), *Inga* *spectabilis* (INSPE), *Miconia* *dissitinervia* (MIDIS), *Miconia* *donaeana* (MIDON), *Miconia* *osaensis* (MIOSA), *Miconia* *trinervia* (MITRI), Ocotea *mollifolia* (OCMOL), *Ocotea* *rivularis* (OCRIV), *Pouteria* *lecytidicarpa* (POLEC), *Pouteria* *subrotata* (POSUB), *Pouteria* *torta* (POTOR), *Pouteria* *triplarifolia* (POTRI), *Protium* *panamense* (PRPAN), *Protium* *pecuniosum* (PRPEC), *Sapium* *allenii* (SAALL), *Sapium* *glandulosum* (SAGLA), *Unonopsis* *osae* (UNOSA), *Unonopsis* *theobromifolia* (UNTHE).
