# Supplementary material for: Is local trait variation related to total range size of tropical trees?
Source: PLoS One. 2018 Mar 7;13(3):e0193268. doi: 10.1371/journal.pone.0193268 (PMC5841763; doi:10.1371/journal.pone.0193268)
Supplement: S6 Table — The functional traits included in the analysis were: Leaf area (LA), leaf thickness (LT), specific leaf area (SLA), leaf dry matter content (LDMC), leaf nitrogen content (N), leaf phosphorus content (P), leaf nitrogen phosphorus ratio (NP) and wood specific gravity (WSG). Statistically significant results (Ho: β = 0) are in bold. (DOCX) [file pone.0193268.s007.docx]

S6 Table. Estimators β ± 1 standard errors of the effects of environmental variables measured and eight functional traits in 335 individual trees of 34 species, according to linear mixed effects models with species as random effect.The functional traits included in the analysis were: Leaf area (LA), leaf thickness (LT), specific leaf area (SLA), leaf dry matter content (LDMC), leaf nitrogen content (N), leaf phosphorus content (P), leaf nitrogen phosphorus ratio (NP) and wood specific gravity (WSG). Statistically significant results (H_0_: β=0) are in bold.

|  |  | **Climatic PC1** | | | | **Crown Light Exposure** | | | | **Inclination of growing sites** | | | |
| --- | --- | --- | --- | --- | --- | --- | --- | --- | --- | --- | --- | --- | --- |
| **Trait** | **DF** | **β** | **Std.Error** | **t.value** | **p.value** | **β** | **Std.Error** | **t.value** | **p.value** | **β** | **Std.Error** | **t.value** | **p.value** |
| LA | 300 | -5.530 | 5.062 | -1.092 | 0.276 | 12.683 | 10.836 | 1.170 | 0.243 | -0.892 | 0.526 | -1.697 | 0.091 |
| LDMC | 300 | 0.199 | 1.113 | 0.179 | 0.858 | **7.806** | **2.339** | **3.338** | **0.001** | -0.149 | 0.116 | -1.288 | 0.199 |
| LT | 300 | **0.003** | **0.001** | **2.580** | **0.010** | **0.007** | **0.003** | **2.593** | **0.010** | 0.000 | 0.000 | -1.894 | 0.059 |
| SLA | 300 | 0.706 | 0.920 | 0.767 | 0.444 | **-7.567** | **1.923** | **-3.935** | **<0.001** | 0.122 | 0.096 | 1.264 | 0.207 |
| N | 287 | 0.001 | 0.009 | 0.116 | 0.908 | 0.009 | 0.020 | 0.460 | 0.646 | 0.000 | 0.001 | -0.149 | 0.882 |
| P | 287 | 0.000 | 0.001 | 0.086 | 0.932 | 0.001 | 0.002 | 0.361 | 0.719 | 0.000 | 0.000 | -0.210 | 0.833 |
| NP | 287 | -0.121 | 0.175 | -0.691 | 0.490 | -0.342 | 0.379 | -0.902 | 0.368 | -0.004 | 0.018 | -0.206 | 0.837 |
| WSG | 298 | **0.005** | **0.002** | **3.143** | **0.002** | 0.003 | 0.004 | 0.883 | 0.378 | 0.000 | 0.000 | -0.130 | 0.896 |
