# Supplementary material for: Is local trait variation related to total range size of tropical trees?
Source: PLoS One. 2018 Mar 7;13(3):e0193268. doi: 10.1371/journal.pone.0193268 (PMC5841763; doi:10.1371/journal.pone.0193268)
Supplement: S7 Table — (DOCX) [file pone.0193268.s008.docx]

S7 Table. Estimated ages for species included in the present analysis according to available phylogenies.

| **Family** | **Species name** | **Range size class** | **Extent of Occurrence** | **Age clade**  **Million years** | **Reference** |
| --- | --- | --- | --- | --- | --- |
| **Annonaceae** | ***Guatteria amplifolia*** | **widespread** | **1.02 • 10^6^** | **1.6 ± 0.6** | **Erkens et al. 2007** |
| **Annonaceae** | ***Guatteria chiriquiensis*** | **endemics** | **8.60 • 10^3^** | **<1.6** | **Erkens et al. 2007** |
| **Annonaceae** | ***Guatteria pudica*** | **endemics** | **6.87 • 10^2^** | **<1.6** | **Erkens et al. 2007** |
| **Annonaceae** | ***Guatteria rostrata*** | **widespread** | **6.45 • 10^4^** | **5-1.6** | **Erkens et al. 2007** |
| **Araliaceae** | ***Dendropanax arboreus*** | **widespread** | **7.69 • 10^6^** | **< 10** | **Li et al. 2013** |
| **Araliaceae** | ***Dendropanax ravenii*** | **endemics** | **1.96 • 10^3^** | **< 10** | **Li et al. 2013** |
| **Burseraceae** | ***Protium panamense*** | **widespread** | **1.98 • 10^5^** | **< 5** | **Fine et al. 2014** |
| **Burseraceae** | ***Protium pecuniosum*** | **endemics** | **1.48 • 10^3^** | **< 5** | **Fine et al. 2014** |

Erkens RHJ, Chatrou LW, Maas JW, van der Niet T, Savolainen V. A rapid diversification of rainforest trees (Guatteria; Annonaceae) following dispersal from Central into South America. Mol Phylogenet Evol. 2007;44: 399–411. doi:10.1016/j.ympev.2007.02.017

Fine PVA, Zapata F, Daly DC. Investigating processes of neotropical rain forest tree diversification by examining the evolution and historical biogeography of the protieae (Burseraceae). Evolution. 2014;68: 1988–2004. doi:10.1111/evo.12414

Li R, Wen J. Phylogeny and Biogeography of Dendropanax (Araliaceae), an Amphi-Pacific Disjunct Genus Between Tropical/Subtropical Asia and the Neotropics. Syst Bot. 2013;38: 536–551. doi:10.1600/036364413X666606
