# Supplementary material for: Is local trait variation related to total range size of tropical trees?
Source: PLoS One. 2018 Mar 7;13(3):e0193268. doi: 10.1371/journal.pone.0193268 (PMC5841763; doi:10.1371/journal.pone.0193268)
Supplement: S8 Table — B: Birds, M: mammals. (DOCX) [file pone.0193268.s009.docx]

S8 Table. Seed size (length x width) and seed dispersers of the 34 neotropical tree species used in the analysis. B: Birds, M: mammals.

| Family | Species name | Seed Size (mm) | Dispersers | Ref. |
| --- | --- | --- | --- | --- |
| Annonaceae | *Guatteria amplifolia* | 10 x 6 | B, M | 1 |
| Annonaceae | *Guatteria pudica* | 8 x 4 | B, M | 1 |
| Annonaceae | *Guatteria rostrata* | 15 x 7 | B, M | 1 |
| Annonaceae | *Guatteria chiriquiensis* | 8 x 4 | B, M | 1 |
| Annonaceae | *Unonopsis osae* | 12 x 6 | B | 2 |
| Annonaceae | *Unonopsis theobromifolia* | 10 x 6 | B | 2 |
| Araliaceae | *Dendropanax arboreus* | 5 x 4 | B | 3 |
| Araliaceae | *Dendropanax ravenii* | 6 x 4 | B | 3 |
| Boraginaceae | *Cordia cymosa* | 9 x 9 | B, M | 4 |
| Boraginaceae | *Cordia liesneri* | 15 x 10 | B | 5 |
| Burseraceae | *Protium panamense* | 13 x 8 | M, B | 6 |
| Burseraceae | *Protium pecuniosum* | 21 x 10 | M, B | 7 |
| Clusiaceae | *Chrysochlamys glauca* | 10 x 4 | B | 6 |
| Clusiaceae | *Chrysochlamys skutchii* | 10 x 4 | B | 8 |
| Clusiaceae | *Garcinia aguilari* | 35 x 35 | M | 8 |
| Clusiaceae | *Garcinia magnifolia* | 30 x 30 | M | 9 |
| Euphorbiaceae | *Sapium allenii* | 5 x 5 | B | 9 |
| Euphorbiaceae | *Sapium glandulosum* | 5 x 5 | B | 9 |
| Fabaceae | *Inga skutchii* | 15 x 5 | M | 10 |
| Fabaceae | *Inga spectabilis* | 35 x 10 | M | 10 |
| Lauraceae | *Ocotea mollifolia* | 40 x 18 | B | 11 |
| Lauraceae | *Ocotea rivularis* | 11 x 6 | B | 11 |
| Melastomataceae | *Miconia dissitinervia* | 0.5 x 0.5 | B | 12 |
| Melastomataceae | *Miconia donaeana* | 0.6 x 0.6 | B | 12 |
| Melastomataceae | *Miconia osaensis* | 0.5 x 0.5 | B | 13 |
| Melastomataceae | *Miconia trinervia* | 0.9 x 0.9 | B | 12 |
| Myrsinaceae | *Ardisia compressa* | 4 x 4 | B | 14 |
| Myrsinaceae | *Ardisia dunlapiana* | 3 x 3 | B | 14 |
| Rubiaceae | *Faramea occidentalis* | 10 x 10 | B | 6 |
| Rubiaceae | *Faramea permagnifolia* | 13 x 13 | B | 15 |
| Sapotaceae | *Pouteria lecytidicarpa* | 29 x 20 | M | 16 |
| Sapotaceae | *Pouteria subrotata* | 22 x 19 | M | 16 |
| Sapotaceae | *Pouteria torta* | 32 x 17 | M | 16 |
| Sapotaceae | *Pouteria triplarifolia* | 30 x 18 | M | 16 |

[1] Maas et al., 2015; [2] Maas et al., 2007; [3] Cannon & Cannon, 1989; [4] Miller, 1987; [5] Miller, 1988; [6] Paton & Calderón, 2016; [7] Daly, 2007; [8] Hammel 2010; [9] González, 2010; [10] Zamora, 2010; [11] González & Hammel, 2007; [12] Almeda, 2007; [13] Kriebel et al., 2008; [14] Morales, 2007; [15] Taylor, 1996; [16] Morales, 2015.

**References**

Almeda, F., 2007. Melastomataceae, in: Hammel, B.E., Grayum, M.H., Herrera, C., Zamora, N. (Eds.), Manual de Plantas de Costa Rica Dicotiledóneas (Haloragaceae-Phytolaccaceae) Vol. VI. Missouri Botanical Garden Press, San Luis, Missouri, pp. 394–574.

Cannon, M.J., Cannon, J.F.M., 1989. Central American araliaceae ― a precursory study for the Flora mesoamericana. Bull. Br. Museum. Nat. Hist. Bot. 19, 5–61.

Daly, D.C., 2007. A new section of Protium from the neotropics . Studies in neotropical Burseraceae xIII. Brittonia 59, 1–24. doi:10.1663/0007-196x(2007)59[1:ANSOPF]2.0.CO;2

González, J., 2010. Euphorbiaceae, in: Hammel, B.E., Grayum, M.H., Herrera, C., Zamora, M. (Eds.), Manual de Plantas de Costa Rica Dicotiledóneas (Clusiaceae - Gunneraceae). Vol. V. Missouri Botanical Garden Press, San Luis, Missouri, pp. 290–394.

González, J., Hammel, B.E., 2007. Ocotea, in: Hammel, B.E., Grayum, M.H., Herrera, C., Zamora, N. (Eds.), Manual de Plantas de Costa Rica Dicotiledóneas (Haloragaceae-Phytolaccaceae) Vol. VI. Missouri Botanical Garden Press, San Luis, Missouri, pp. 129–158.

Hammel, B.E., 2010. Clusiaceae, in: Hammel, B.E., Grayum, M.H., Herrera, C., Zamora, N. (Eds.), Manual de Plantas de Costa Rica Dicotiledóneas (Clusiaceae - Gunneraceae). Vol. V. Missouri Botanical Garden Press, San Luis, Missouri, pp. 1–54.

Kriebel, R., Aguilar, R., Almeda, F., 2008. A New and threatened arborescent Miconia ( Melastomataceae : Miconieae ) from the Osa Peninsula , Costa Rica. Proc. Calif. Acadamy Sci. 59, 489–495.

Maas, P.J.M., Westra, L.Y.T., Arias Guerrero, S., Lobão, A.Q., Scharf, U., Zamora, N.A., Erkens, R.H.J., 2015. Confronting a morphological nightmare: Revision of the Neotropical genus *Guatteria* (Annonaceae). Blumea J. Plant Taxon. Plant Geogr. 60, 1–219. doi:10.3767/000651915x690341

Maas, P.J.M., Westra, L.Y.T., Vermeer, M., 2007. Revision of the Neotropical genera Bocageopsis, Onychopetalum, and Unonopsis (Annonaceae). Blumea 52, 413–554.

Miller, J.S., 1988. A Revised Treatment of Boraginaceae for Panama. Ann. Missouri Bot. Gard. 75, 456. doi:10.2307/2399433

Miller, J.S., 1987. Two New Species of Cordia (Boraginaceae) from Central America. Ann. Missouri Bot. Gard. 74, 670. doi:10.2307/2399333

Morales, J.F., 2007. Myrsinaceae, in: Hammel, B.E., Grayum, M.H., Herrera, C., Zamora, N. (Eds.), Manual de Plantas de Costa Rica Dicotiledóneas (Haloragaceae-Phytolaccaceae) Vol. VI. Missouri Botanical Garden Press, San Luis, Missouri, pp. 692–727.

Morales, J.F., 2015. Sapotaceae., in: Hammel, B.E., Grayum, M.H., Herrera, C., Zamora, N. (Eds.), Manual de Plantas de Costa Rica. Dicotiledóneas (Sabiaceae - Zygophyllaceae) Vol. VIII.Vol. VIII. Missouri Botanical Garden Press, pp. 96–140.

Taylor, C.M., 1996. More New Species and a New Combination in Rubiaceae from Costa Rica and Panama. Novon 6, 298. doi:10.2307/3392098

Paton, S., Calderón, O., 2016. Plant Images Database. [WWW Document]. URL http://www.stri.si.edu/sites/esp/tesp/plant_images_info.htm (accessed 12.12.16).

Zamora, N., 2010. Fabaceae, in: Hammel, B.E., Grayum, M.H., Herrera, C., Zamora, N. (Eds.), Manual de Plantas de Costa Rica Dicotiledóneas (Clusiaceae - Gunneraceae). Vol. V. Missouri Botanical Garden Press, San Luis, Missouri, pp. 395–775.
