# Supplementary figures and images for: Is local trait variation related to total range size of tropical trees?
Source: PLoS One. 2018 Mar 7;13(3):e0193268. doi: 10.1371/journal.pone.0193268 (PMC5841763; doi:10.1371/journal.pone.0193268)

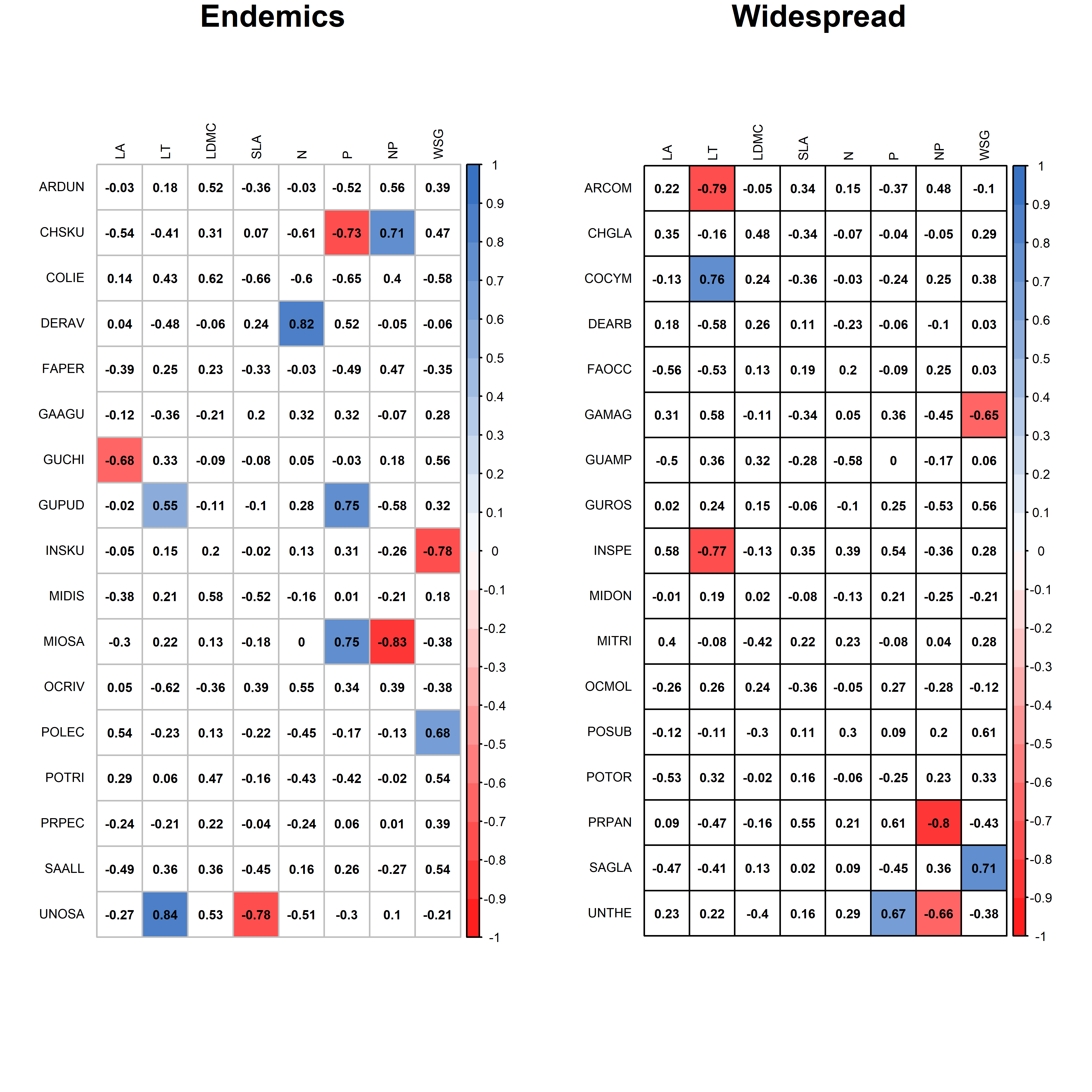

Supplement: S1 Fig — Correlation coefficients significantly different from zero (p<0.05, 20 out of 272) are presented with color. (TIFF) [file pone.0193268.s010.tiff]

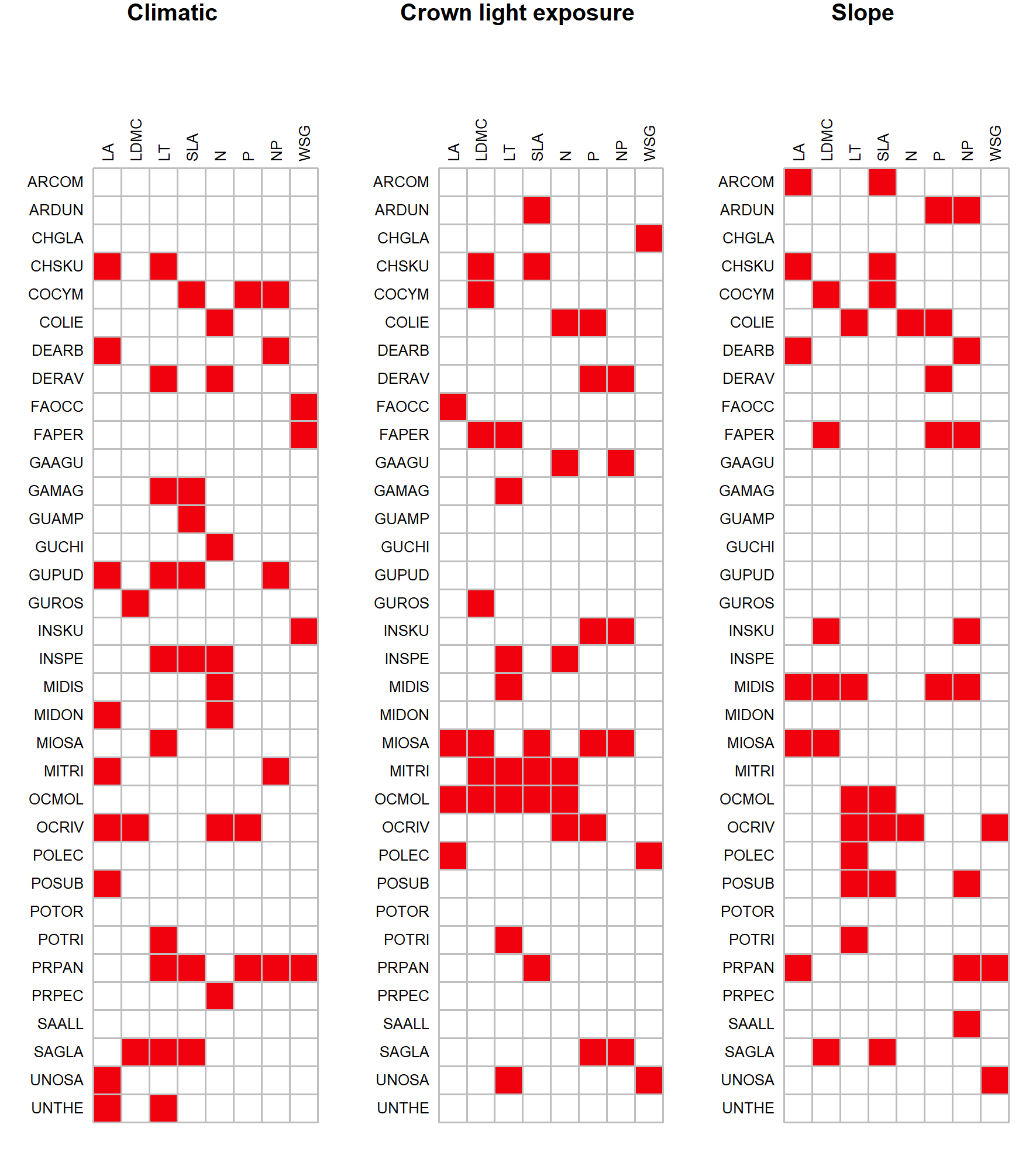

Supplement: S2 Fig — Red squares indicate models with p-value< 0.1. Functional traits are abbreviated as follows: leaf area (LA), leaf dry matter content (LDMC), leaf thickness (LT), specific leaf area (SLA), leaf nitrogen content (N), leaf phosphorus content (P), leaf nitrogen to phosphorus ratio (N:P) and wood specific gravity (WSG). Species codes are in S5 Table. (TIFF) [file pone.0193268.s011.tiff]

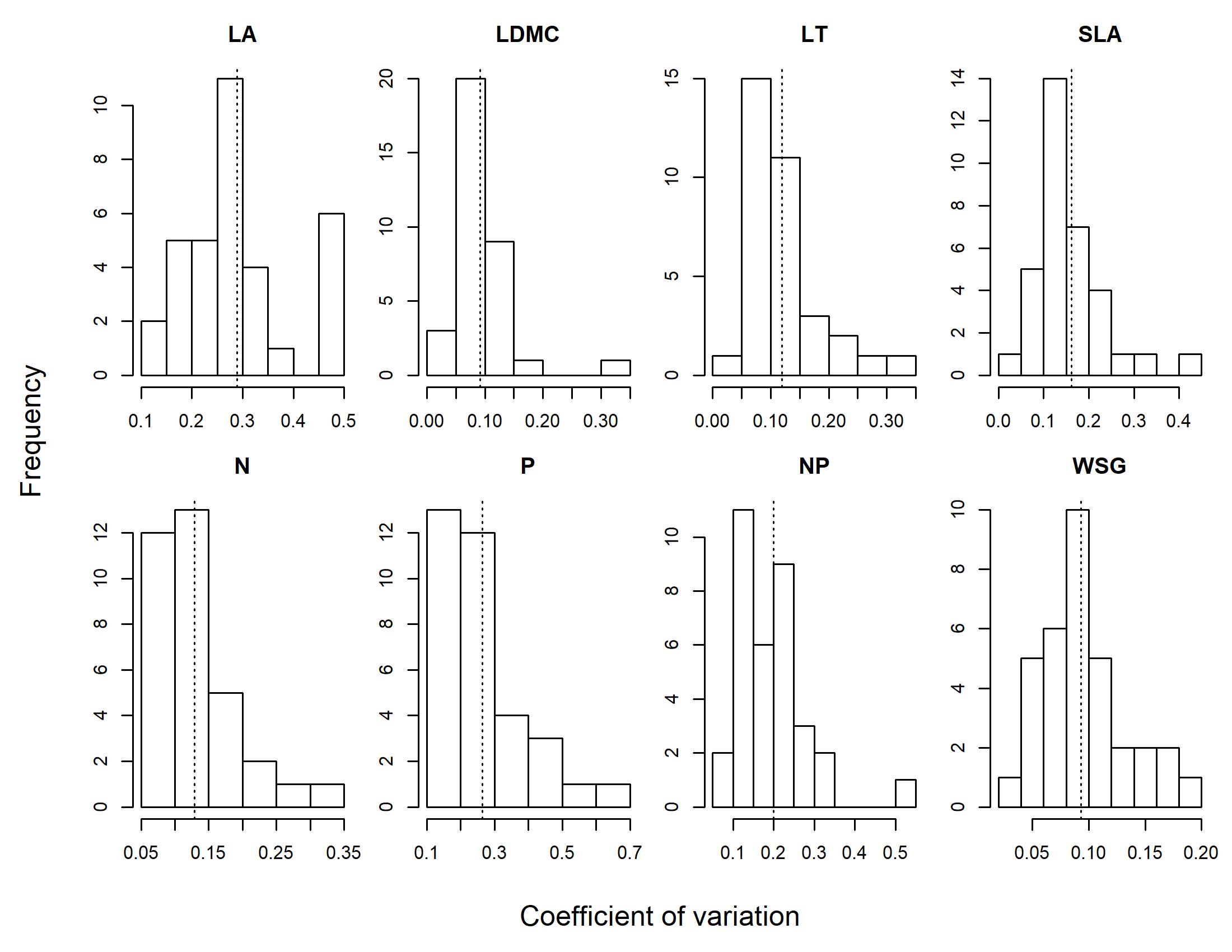

Supplement: S3 Fig — The dotted vertical line indicates the mean of the coefficients of variation. Leaf area (LA), leaf dry matter content (LDMC), leaf thickness (LT), specific leaf area (SLA), leaf nitrogen content (N), leaf phosphorus content (P), leaf N:P ratio (NP) and wood specific gravity (WSG). (TIFF) [file pone.0193268.s012.tiff]

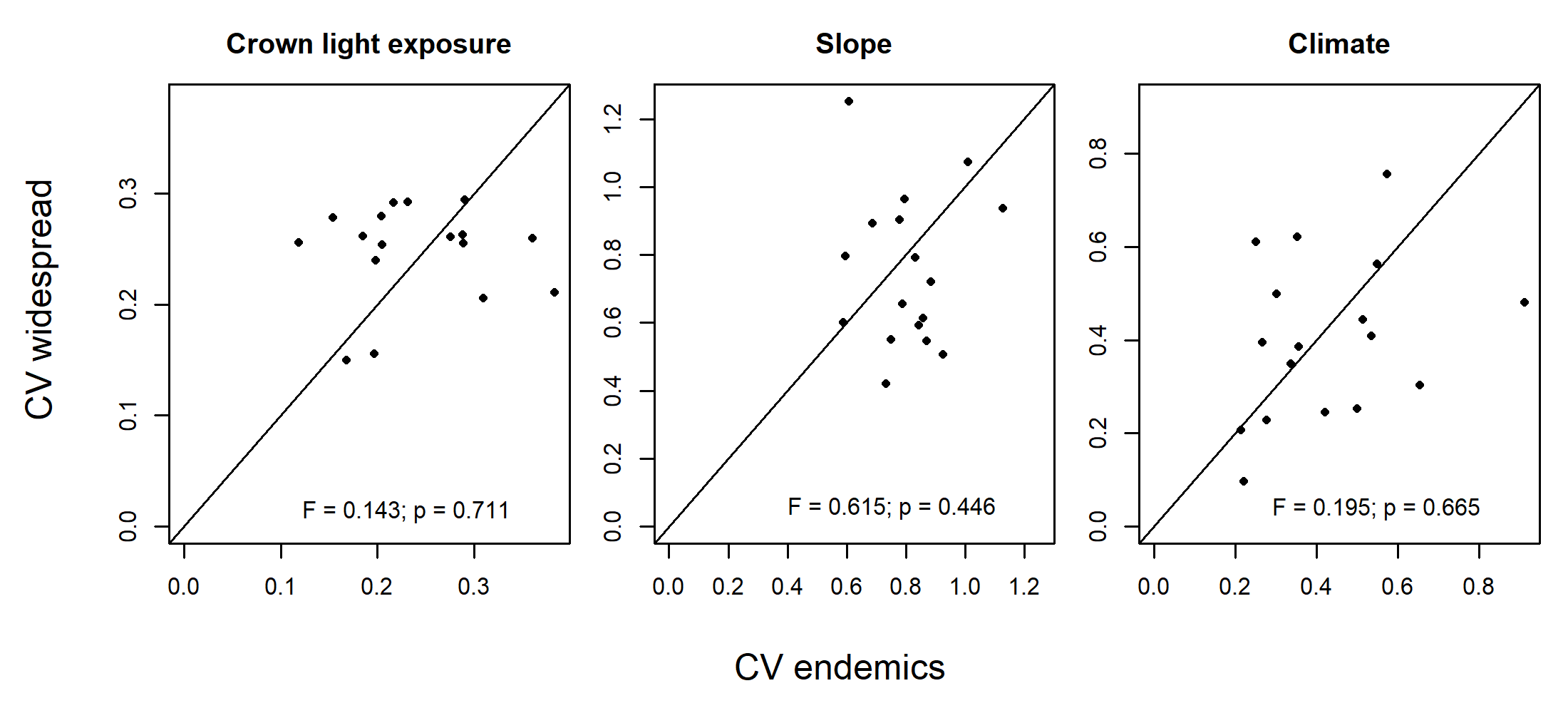

Supplement: S4 Fig — Each point represents one pair of congeneric endemic and widespread species. The diagonal represents the null model, i.e. positioning of points along the line indicates equal environmental variability among the sampled trees of both species in a pair. Points above the line represent pairs with environmental CV higher in widespread species and points below the line pairs with CV higher in endemic species. (TIFF) [file pone.0193268.s013.tiff]

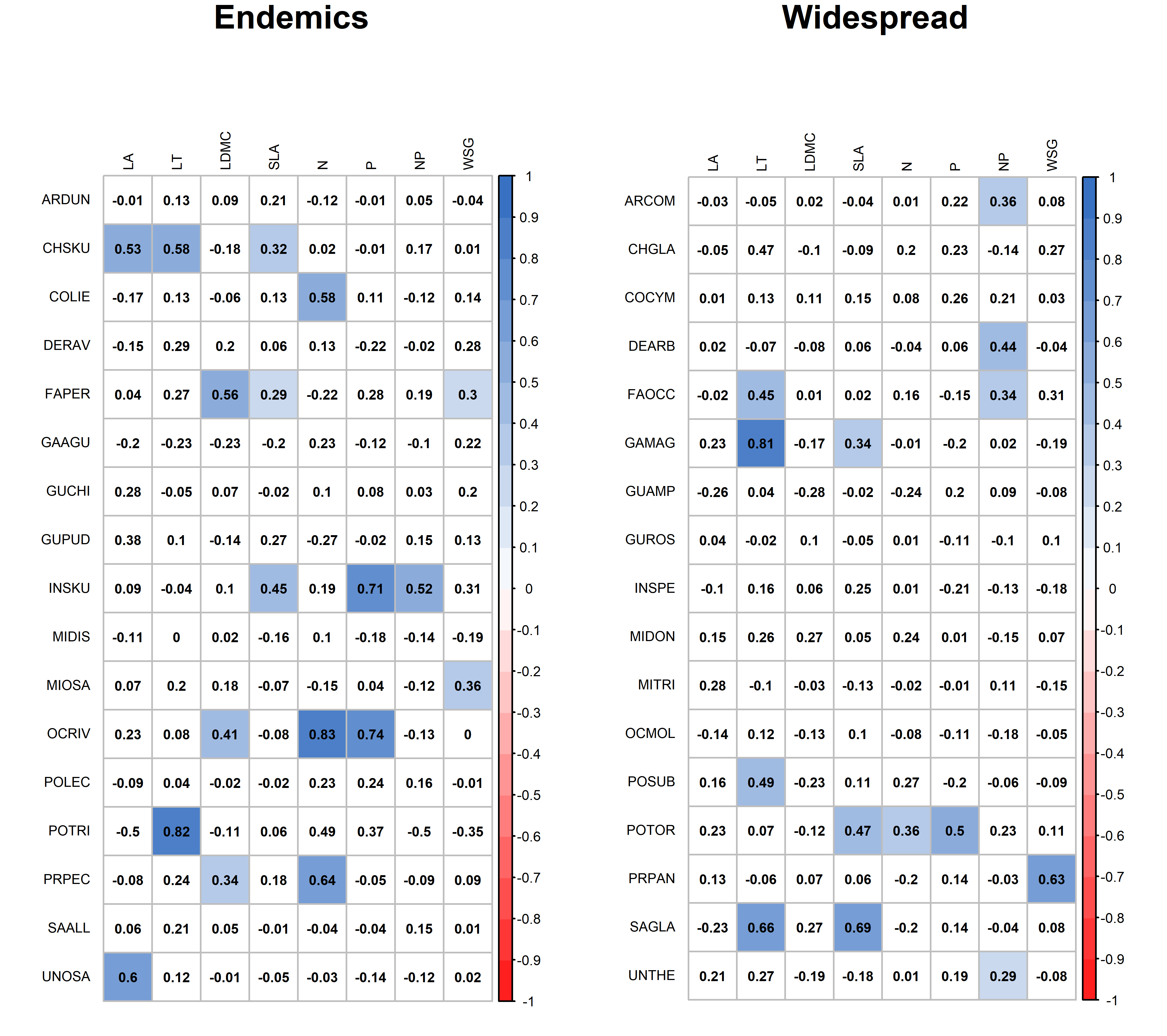

Supplement: S5 Fig — Correlation coefficients significantly different from zero (p<0.05) are presented with color. (TIFF) [file pone.0193268.s014.tiff]

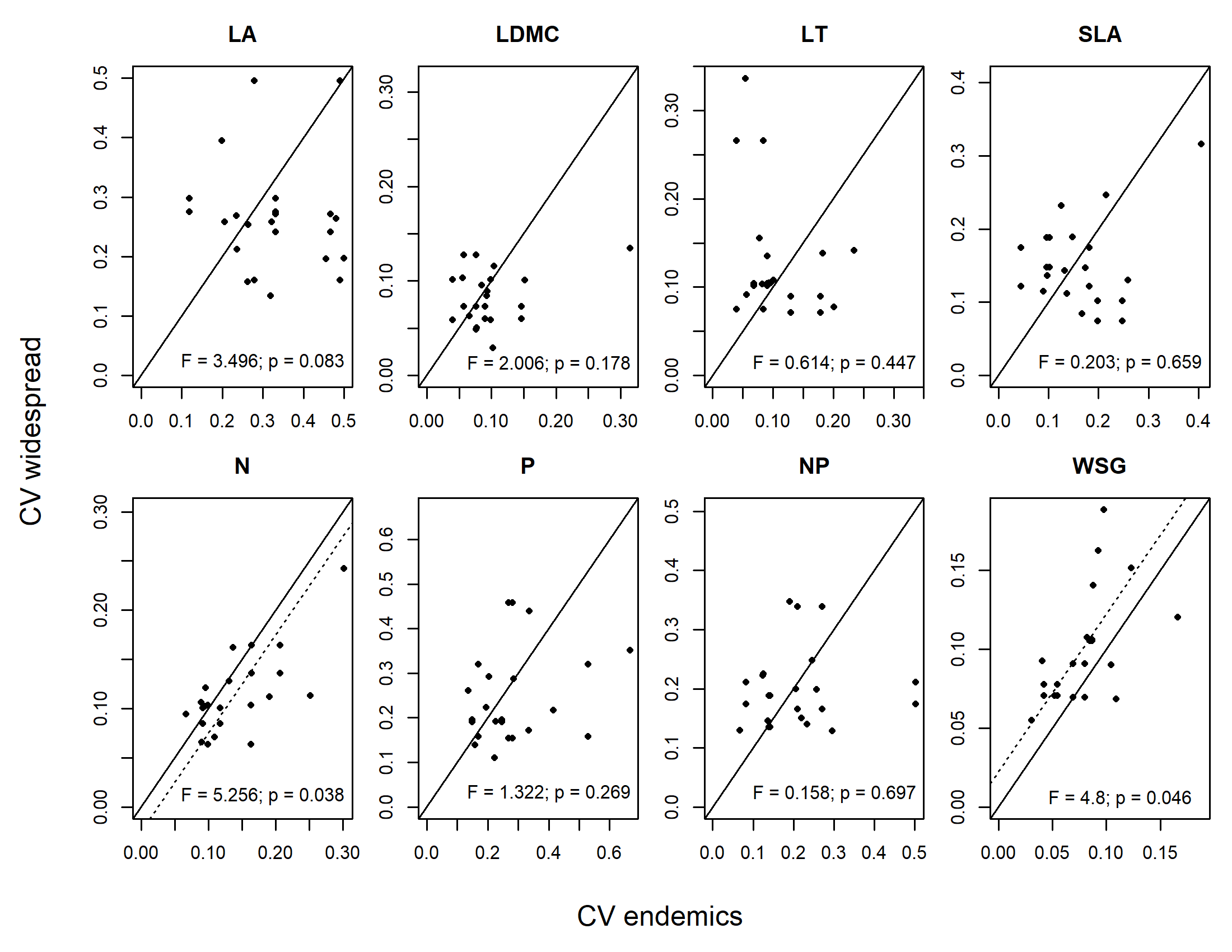

Supplement: S6 Fig — (TIFF) [file pone.0193268.s015.tiff]

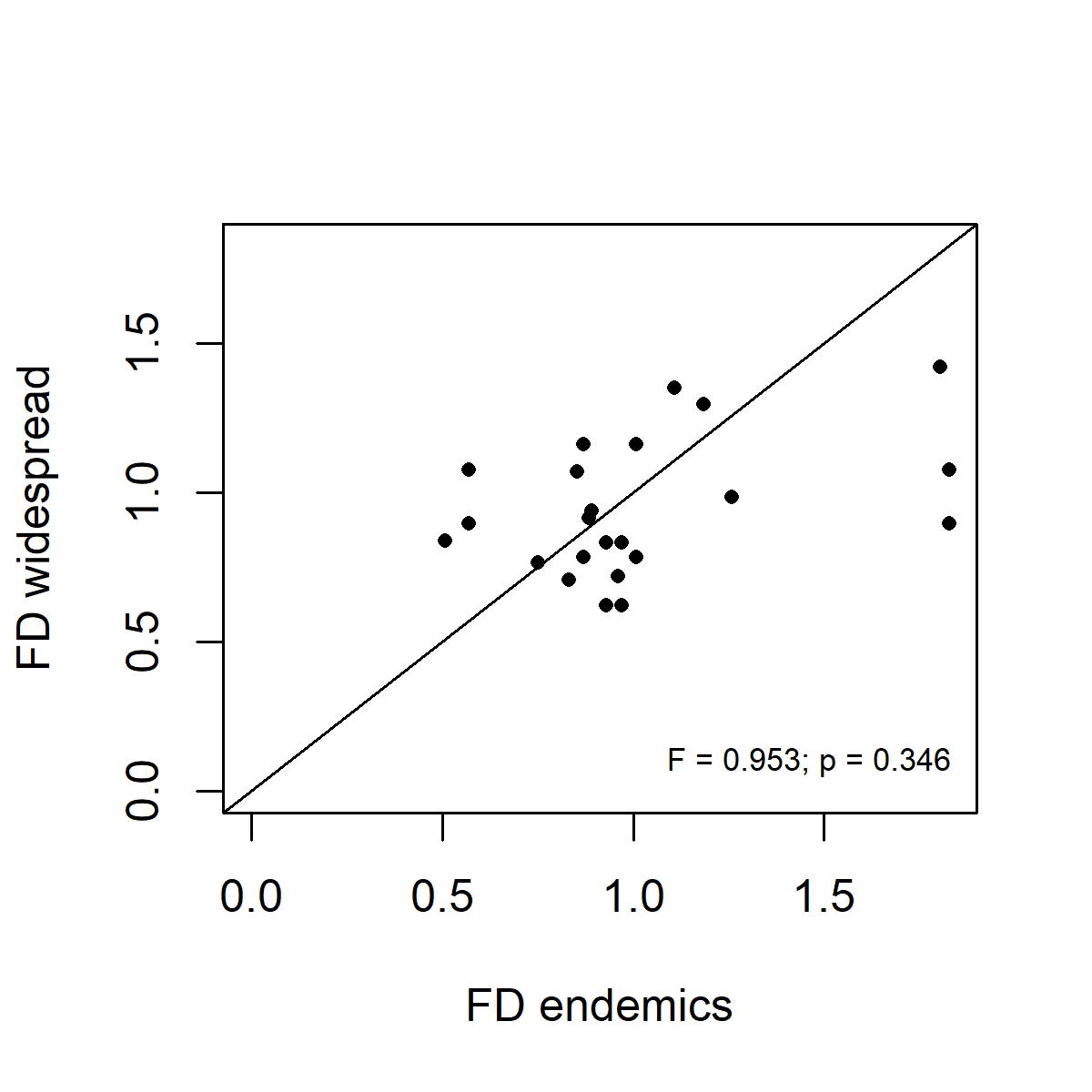

Supplement: S7 Fig — (TIFF) [file pone.0193268.s016.tiff]

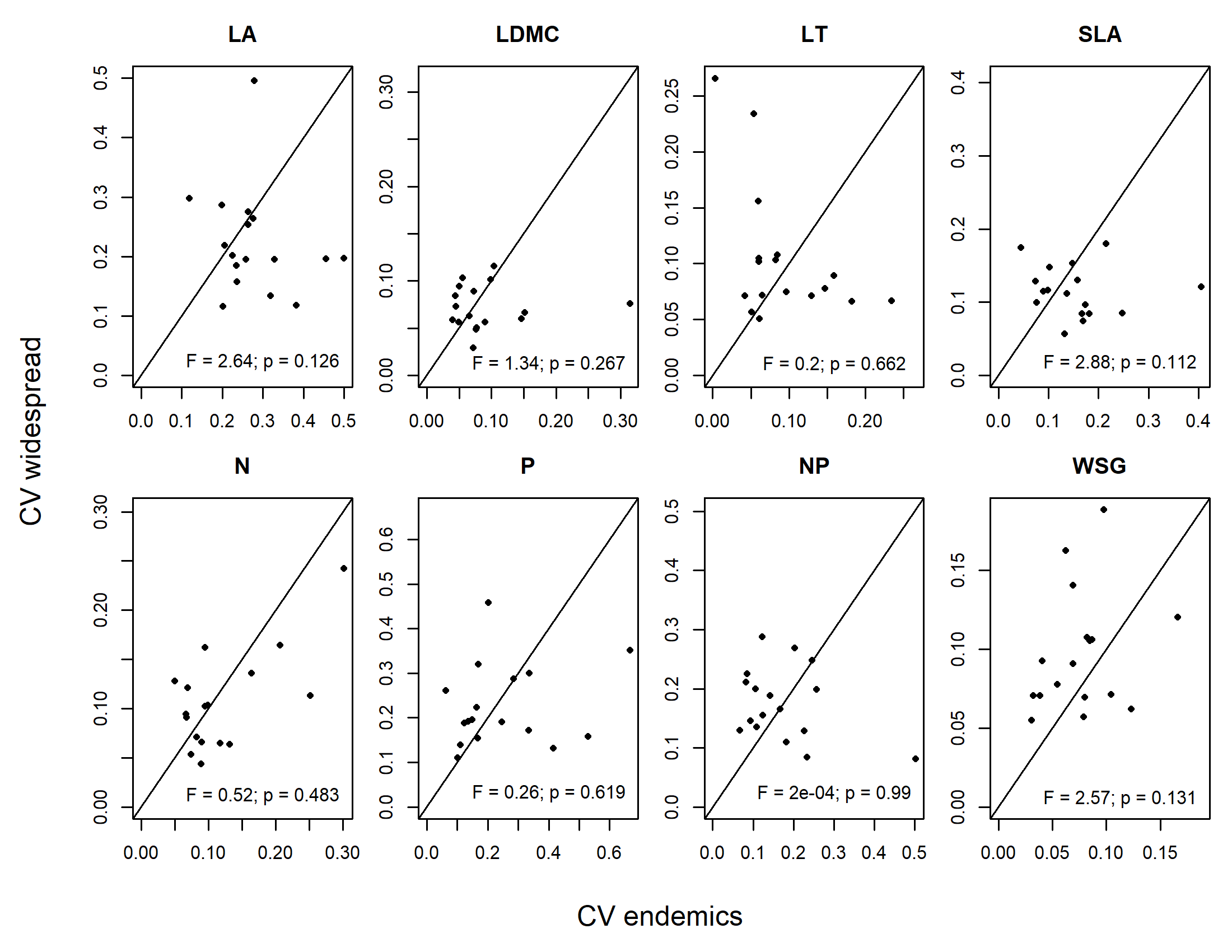

Supplement: S8 Fig — Leaf area (LA), leaf thickness (LT), specific leaf area (SLA) and leaf dry matter content (LDMC), wood specific gravity (WSG), leaf nitrogen content (N) and leaf phosphorus content (P), and N:P ratio of endemic neotropical tree species and their widespread congeners after removing variation related to variation in environmental variables. Each point represents one congeneric pair of species (endemic, widespread). The diagonal represents the null model, i.e. positioning of points along the line indicates equal trait variability of both species in a pair. Points above the line represent pairs with CV greater in widespread species, and points below the line pairs with CV greater in endemic species. The p-value is for the associated statistics testing if the intercept = 0. (TIFF) [file pone.0193268.s017.tiff]

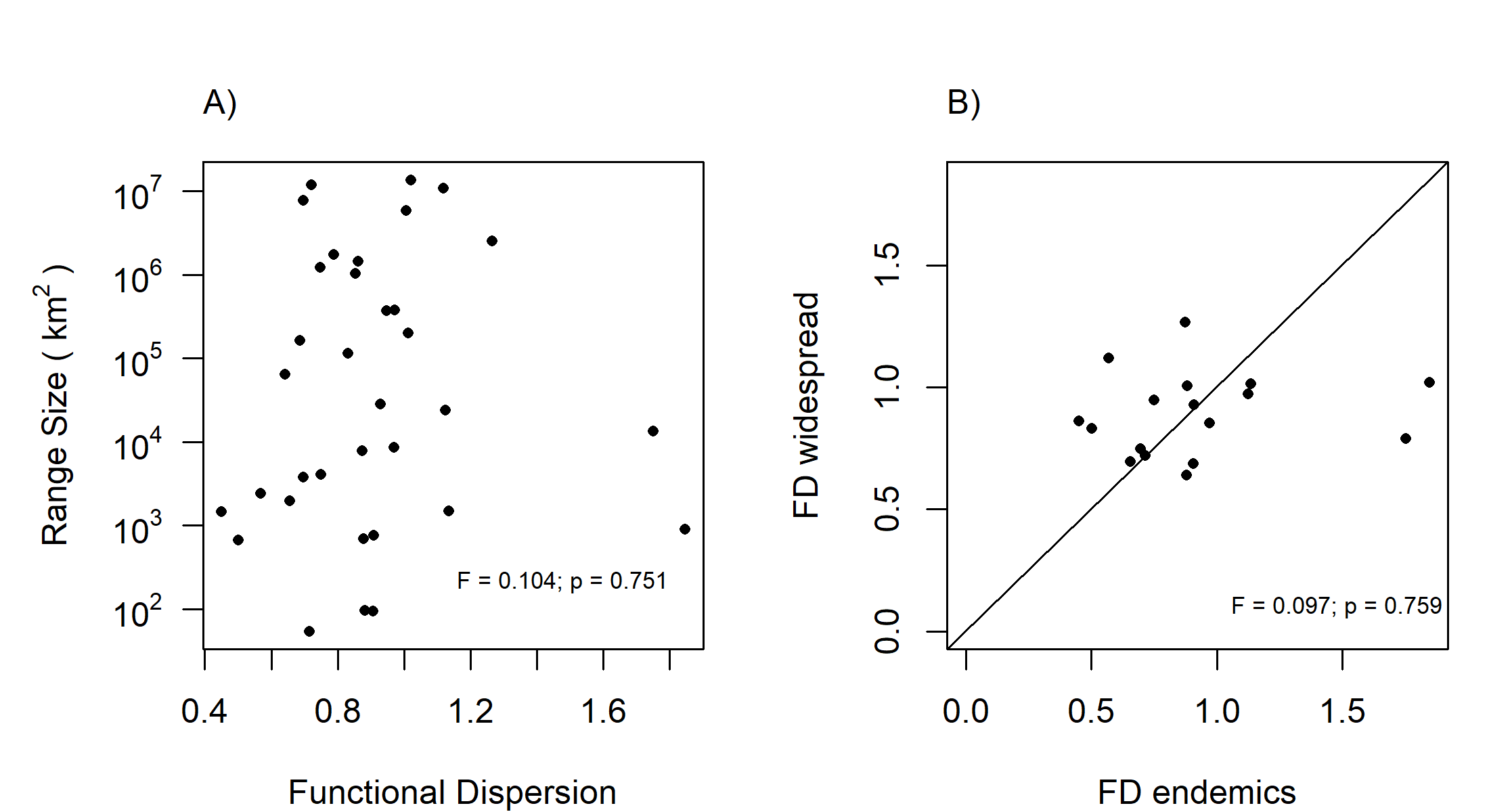

Supplement: S9 Fig — A) Functional dispersion in relation to the range size. B) Functional dispersion (FD) of 17 congeneric pairs of endemic species and their widespread congeners. In Fig 5B each point represents one pair (endemic, widespread). The diagonal represents the null model, i.e. positioning of points along the line indicates equal functional dispersion of both species in a pair. Points above the line represent pairs with FD higher in widespread species, and points below the line pairs with FD higher in endemic species. (TIFF) [file pone.0193268.s018.tiff]

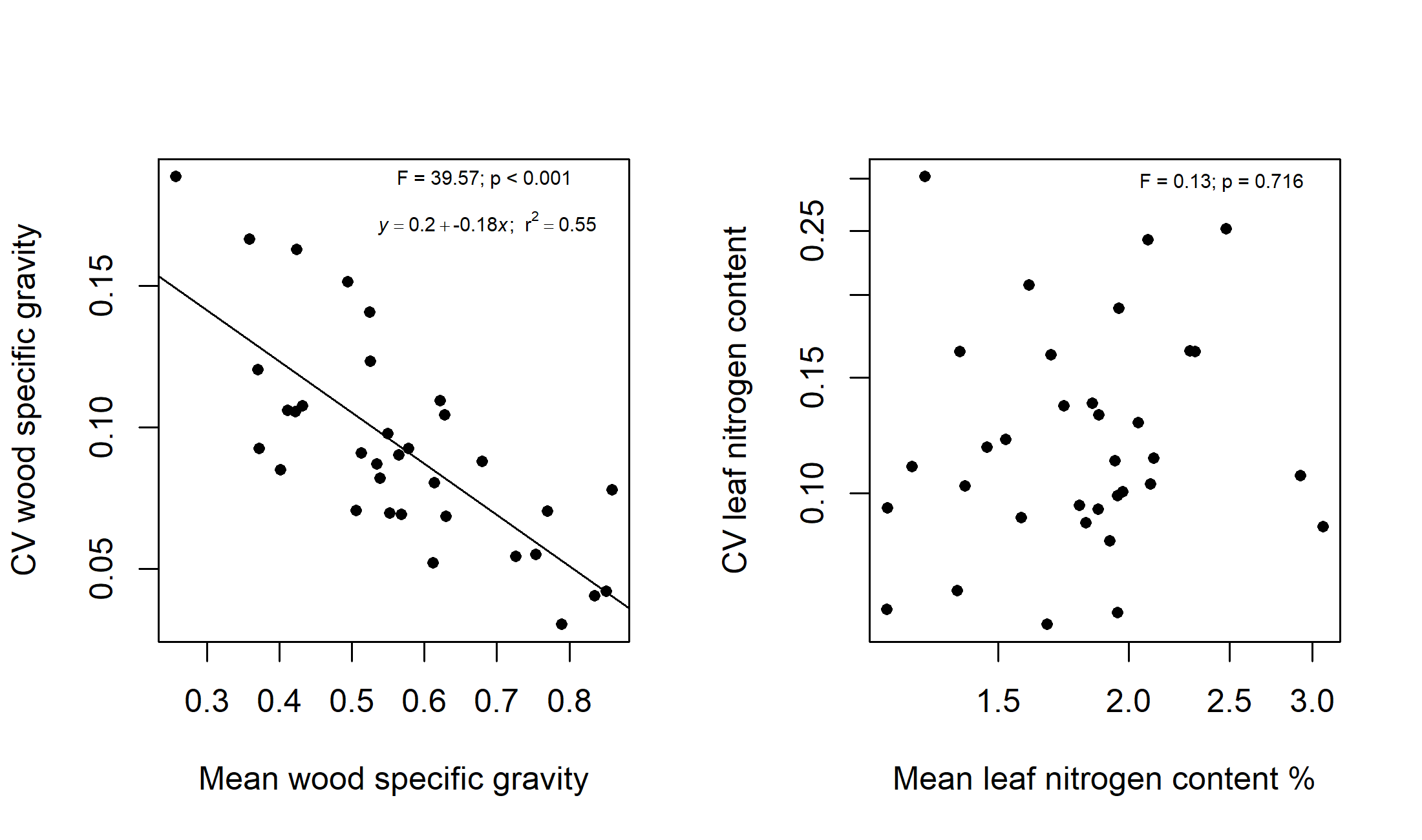

Supplement: S10 Fig — The regression line is represented by a solid line when the effect of the regressor was significantly different from zero, and with a dotted line when it was not significant. (TIFF) [file pone.0193268.s019.tiff]
