# Supplementary material for: Is local trait variation related to total range size of tropical trees?
Source: PLoS One. 2018 Mar 7;13(3):e0193268. doi: 10.1371/journal.pone.0193268 (PMC5841763; doi:10.1371/journal.pone.0193268)

S1 File. Maps of the geographical ranges of the 34 tree species studied. Maps represent occurrences derived from different sources plotted as red points. The green polygon was constructed using an alpha-hull algorithm with 8 as alpha value. Maps in the same row belong to species in the same genus, maps on the right side are for endemic species and maps on the left side are for widespread species. Below the map is the amount of records available for each species.


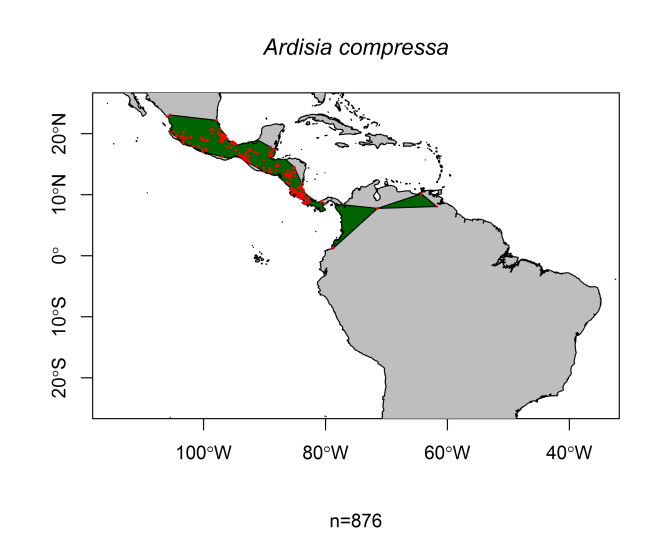

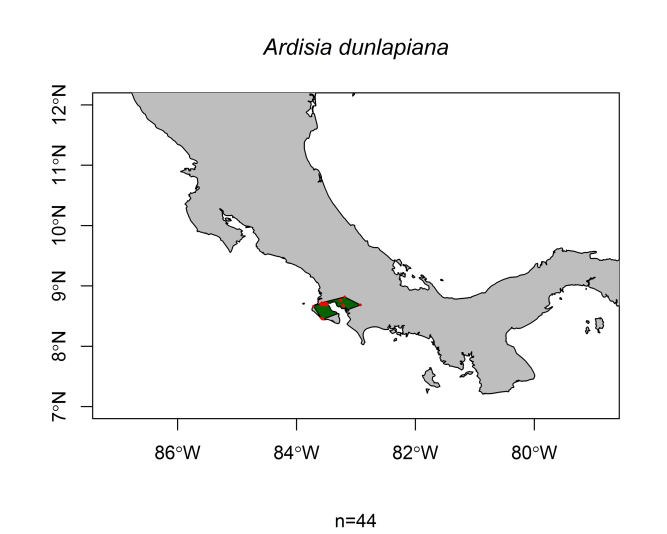


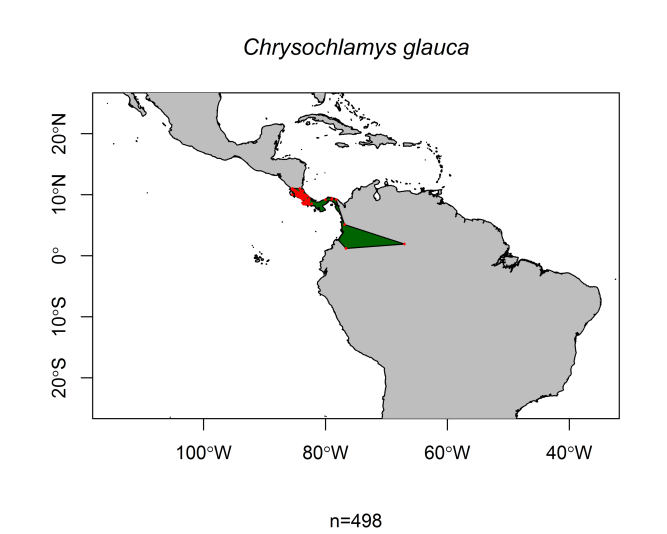

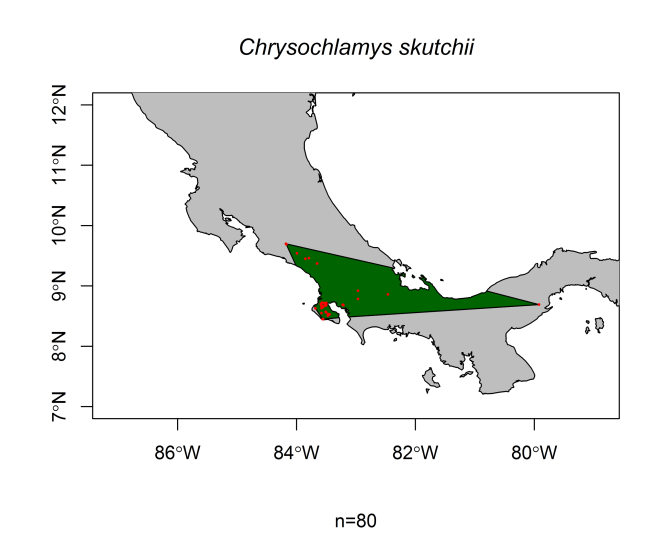

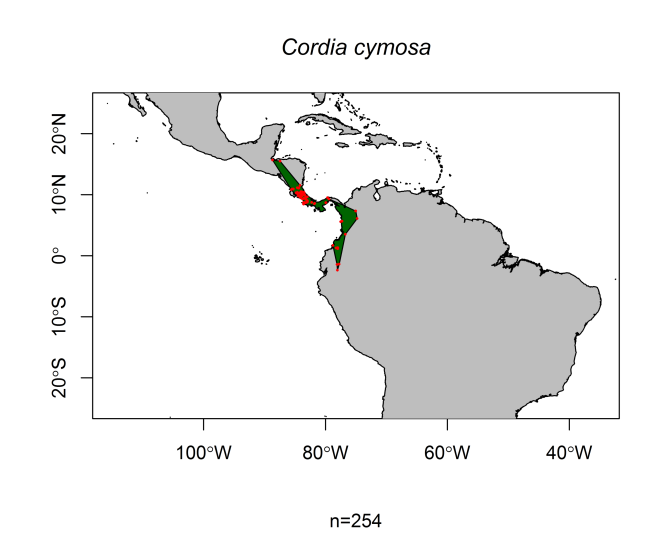

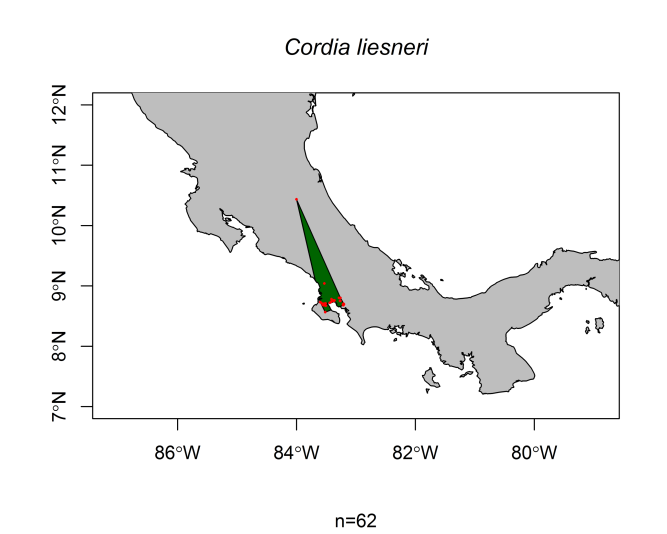

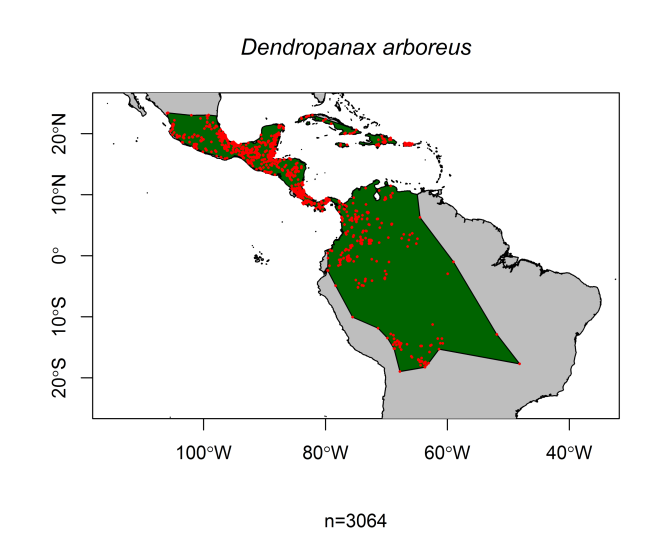

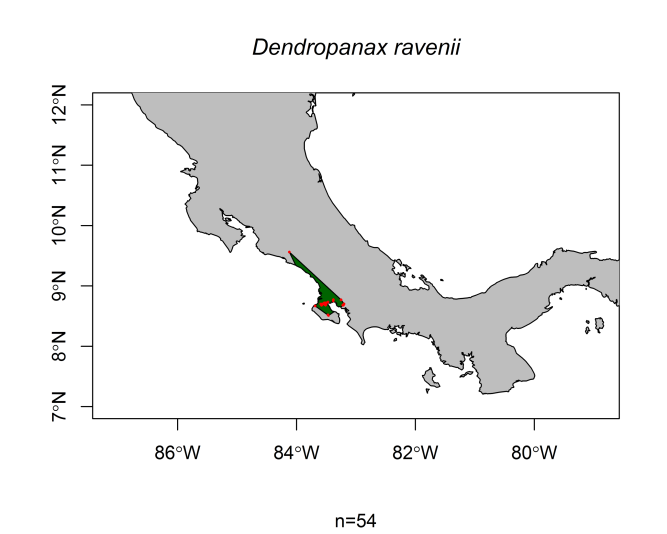

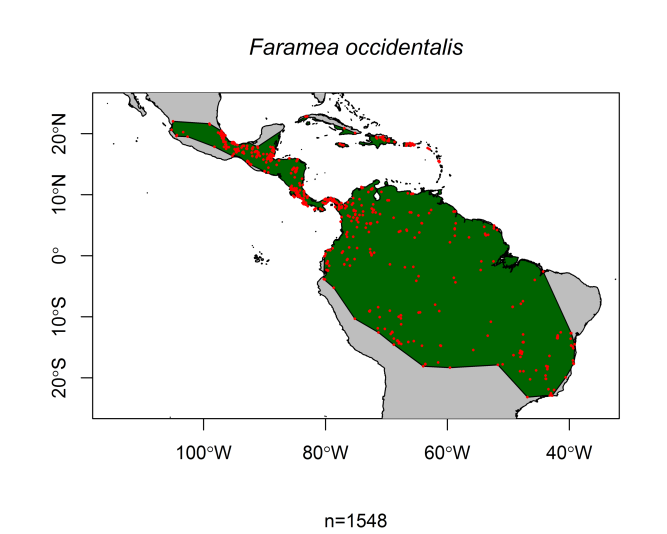

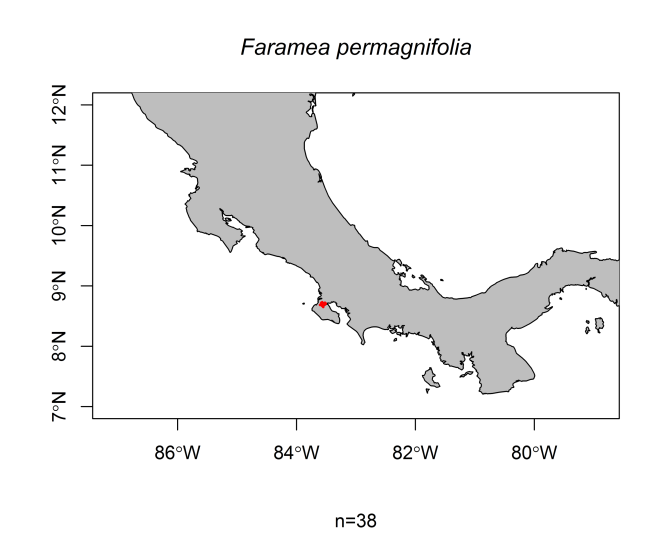


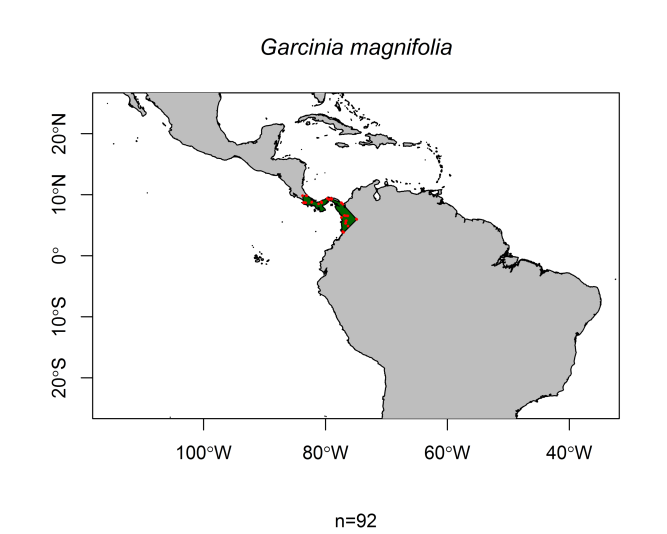

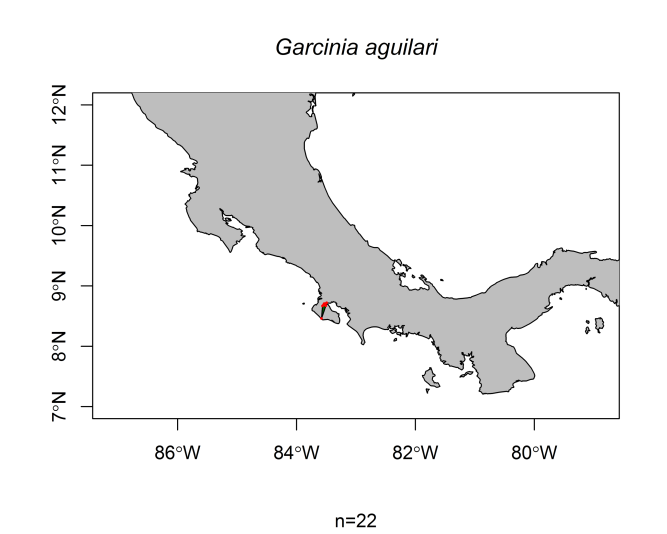


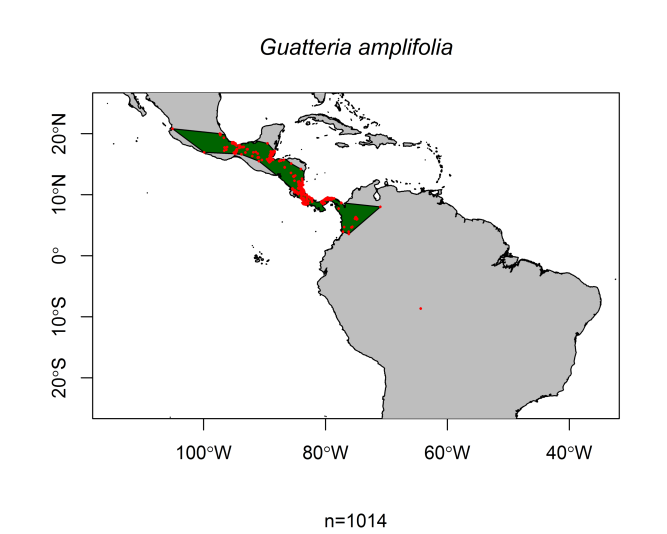

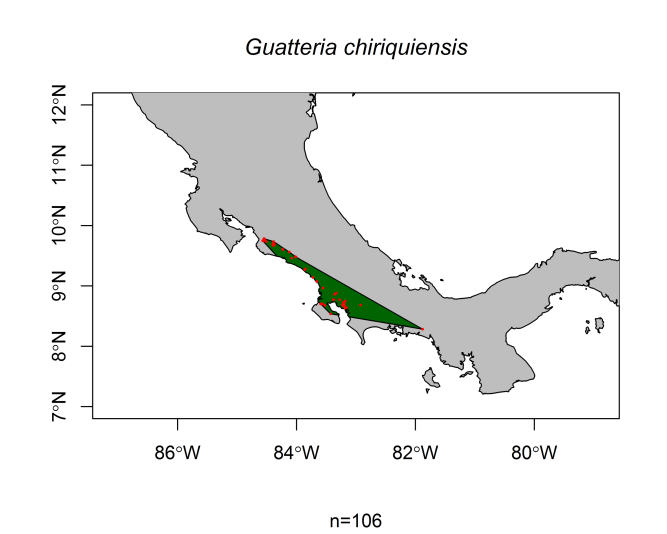


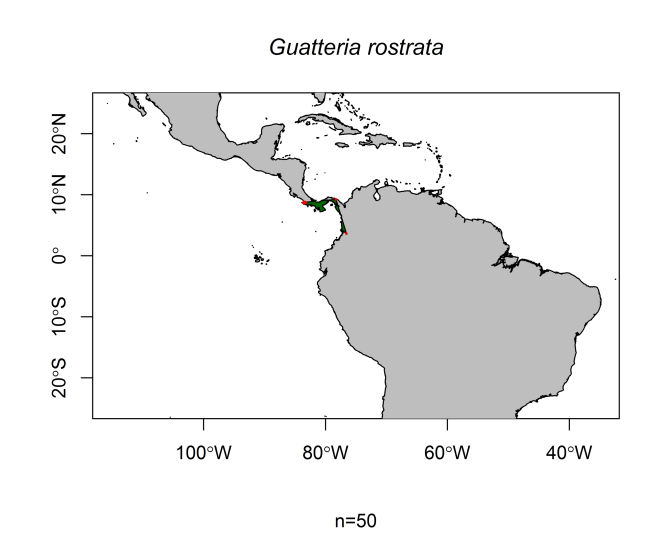

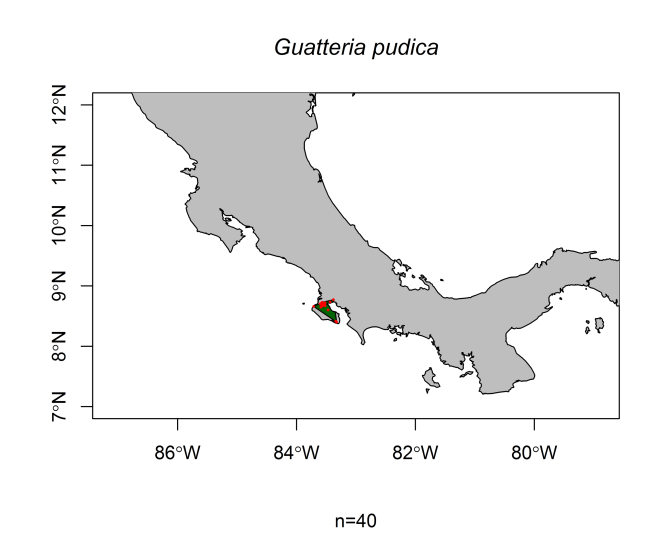

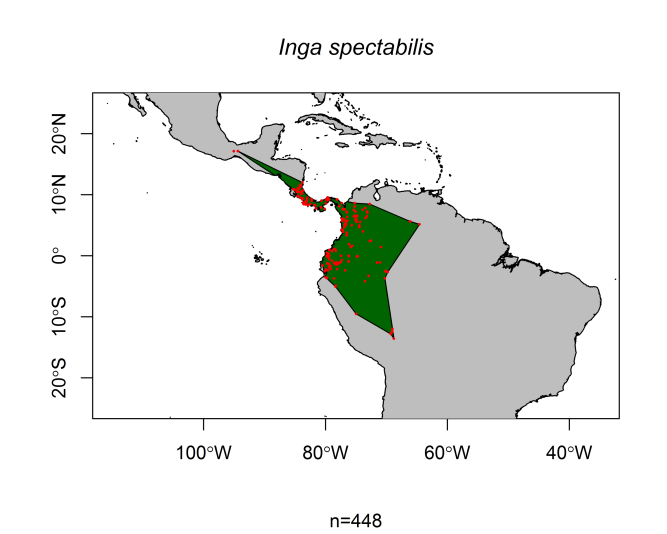

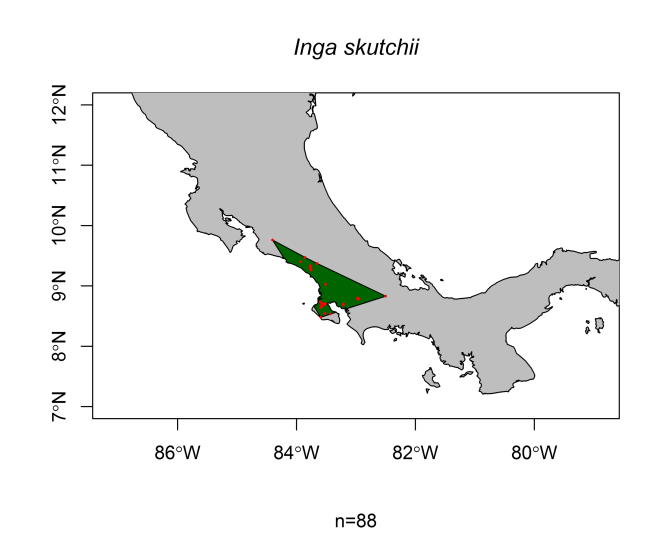


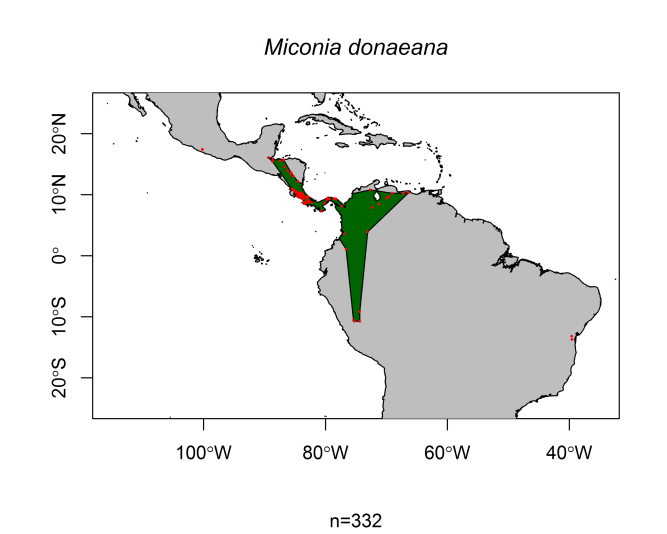

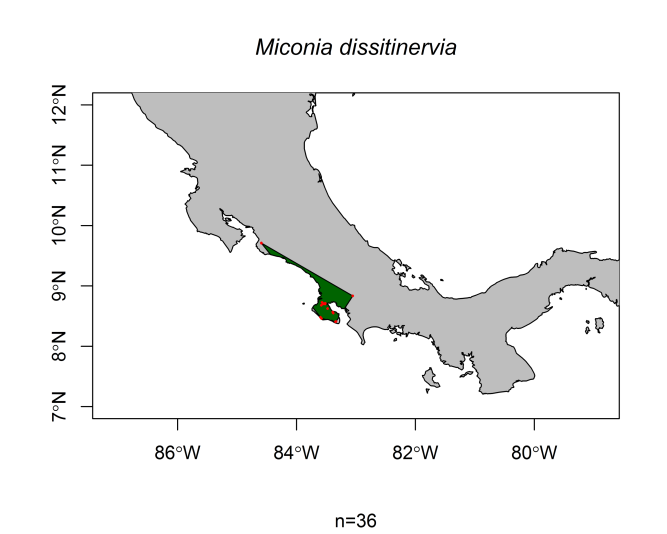


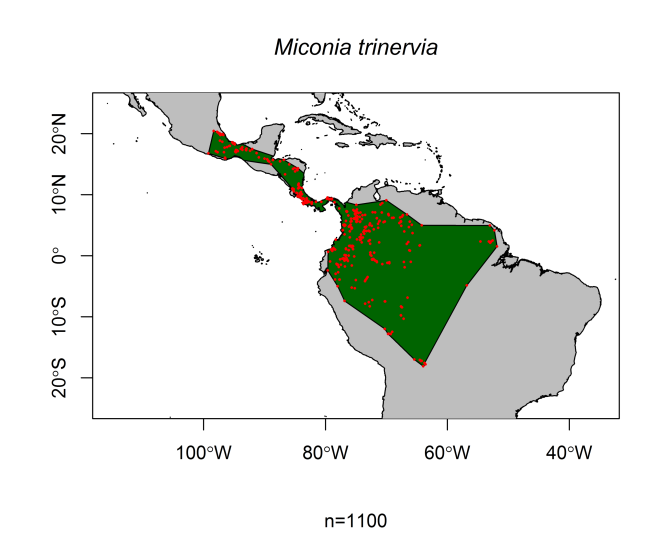

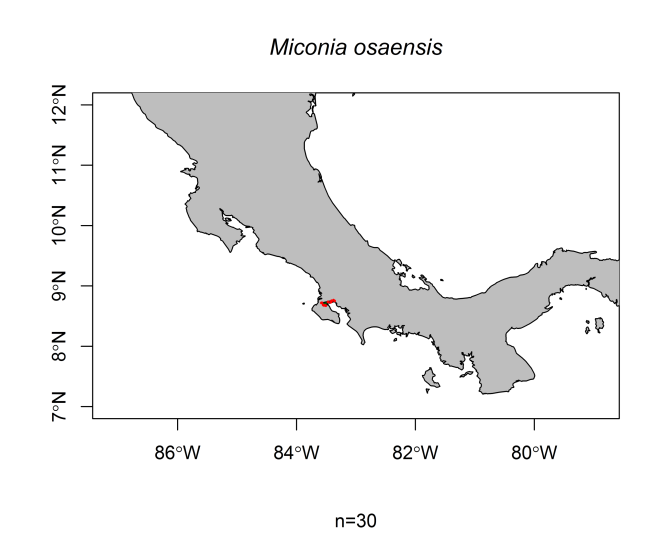


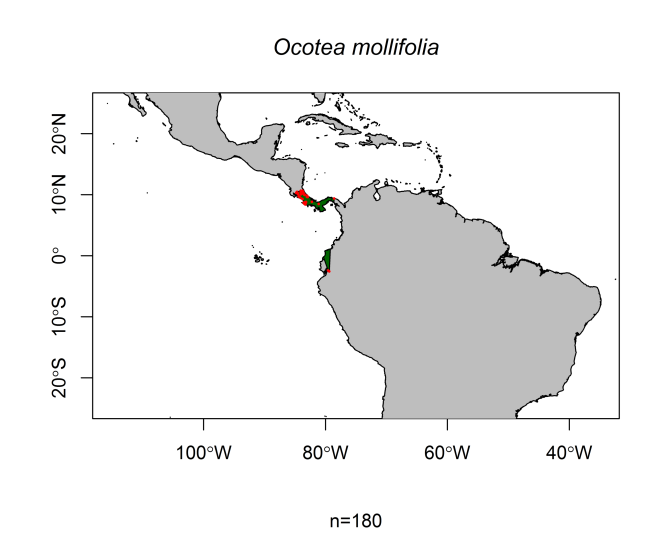

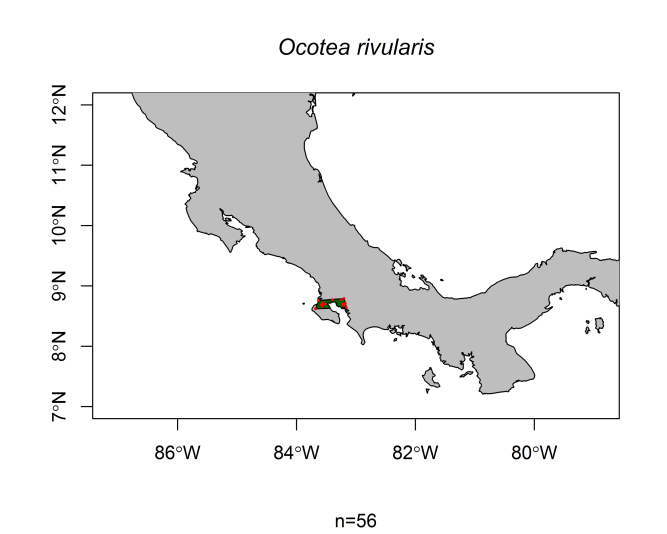


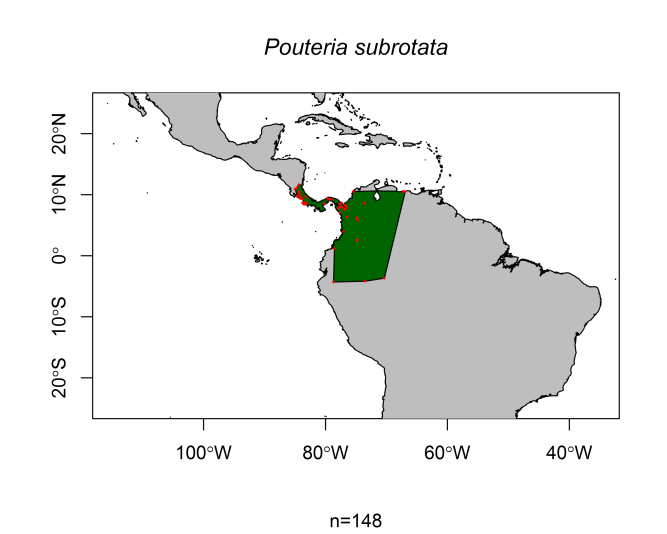

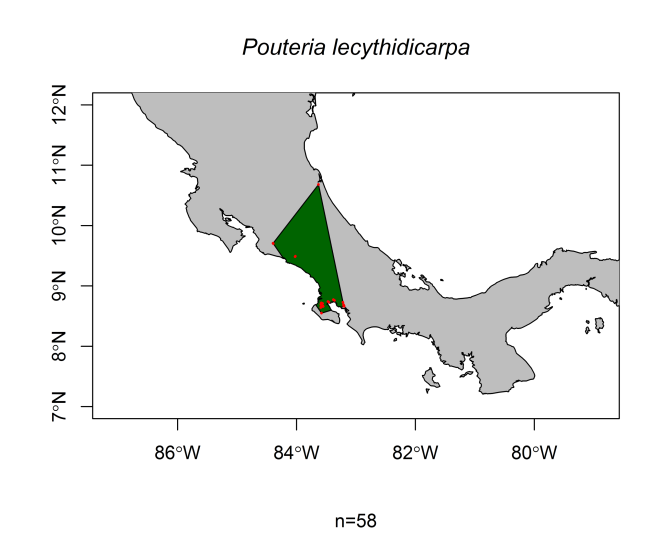

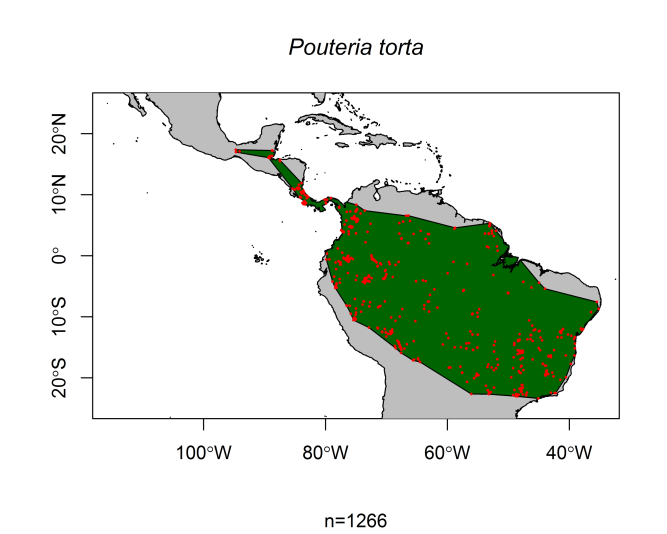

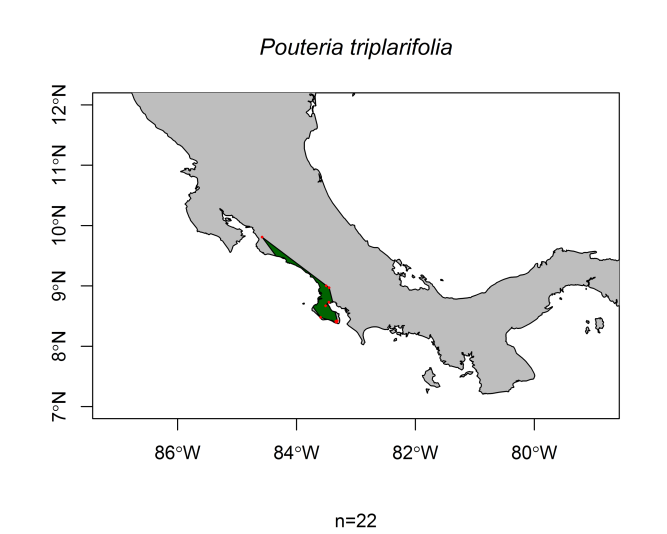

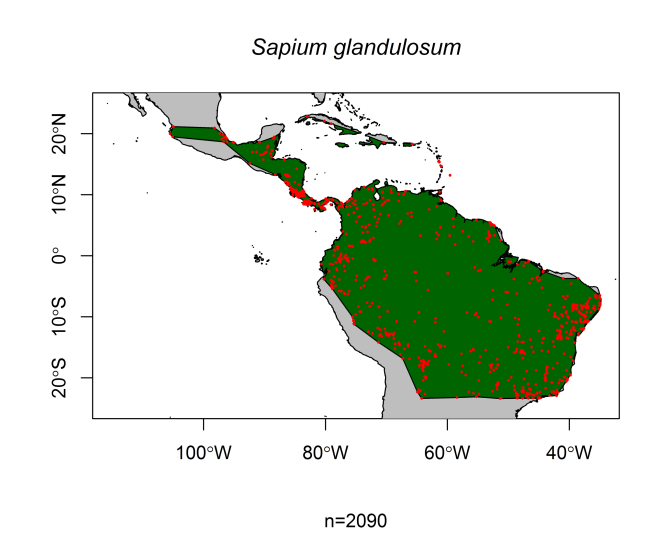

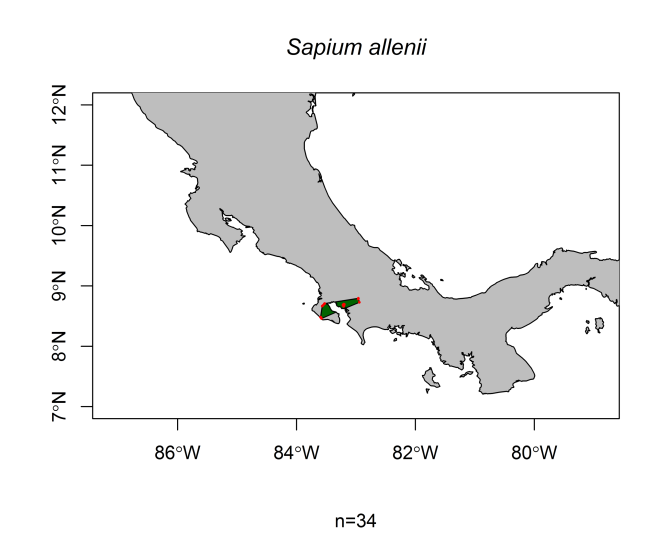


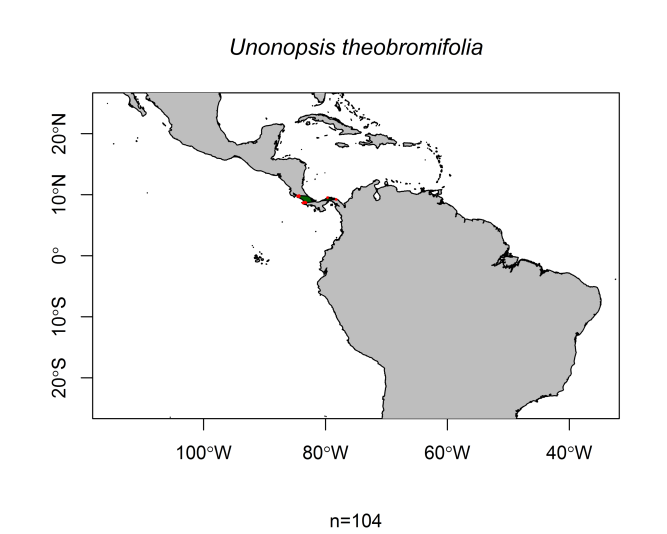

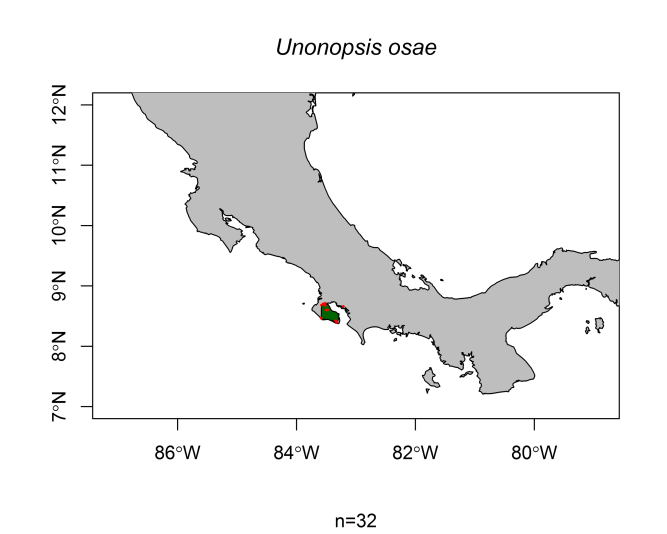

Supplement: S1 File — (DOCX) [file pone.0193268.s020.docx]
